# Enhanced resistance of metal sequestering agents by reconfiguration of the *Staphylococcus aureus* cell wall

**DOI:** 10.1038/s44259-025-00131-1

**Published:** 2025-07-03

**Authors:** Joy R. Paterson, Joshua M. Wadsworth, Rebecca J. Lee, Ping Hu, Jacob Biboy, Daniela Vollmer, Waldemar Vollmer, Jon Marles-Wright, Jana N. Radin, Thomas E. Kehl-Fie, Mary T. Moran, Gary J. Sharples

**Affiliations:** 1 Department of Biosciences, Durham University, Durham, UK; 2 Procter and Gamble, Mason Business Center, Cincinnati, OH USA; 3 Centre for Bacterial Cell Biology, Biosciences Institute, Newcastle University, Newcastle upon Tyne, UK; 4 Institute for Molecular Bioscience, The University of Queensland, Brisbane, QLD Australia; 5 Biosciences Institute, Newcastle University, Newcastle upon Tyne, UK; 6 Department of Microbiology and Immunology, University of Iowa, Iowa City, IA USA; 7 Procter & Gamble Technical Centres, Reading, Berkshire, UK

**Keywords:** Antimicrobial resistance, Antimicrobials, Experimental evolution

## Abstract

Chelators possess antibacterial properties linked to metal sequestration, simulating the action of nutritional immunity in preventing infection. To gain further insight into bacterial adaptation to metal restriction, we isolated mutants of *Staphylococcus aureus* with enhanced resistance to two synthetic chelators with therapeutic potential. Mutations were identified that altered peptidoglycan metabolism and teichoic acid modification, crucially affecting PBP2 and eliminating FmtA or VraF functionality. The resulting strains showed increased cell wall thickness, modified cell surface charge and varied in susceptibility to cell wall-targeting agents. In those mutants lacking either FmtA or VraF, the modifications substantially increased cell surface-associated calcium, offering protection against loss of manganese that was preferentially targeted by both chelators. Our phenotypic and cellular metal analyses identify the cell envelope of *S. aureus* as a key target for metal sequestering molecules. Peptidoglycan and teichoic acids, in particular, serve as key repositories for a subset of metal ions that safeguard against deprivation and can be altered to augment resistance to antibacterial chelators.

## Introduction

Alternatives to antibiotics are urgently needed to combat microbes that rapidly develop resistance to existing drug regimens^1^. An estimated 1.27 million deaths worldwide were attributed to bacterial antimicrobial resistance in 2019^2^, predicted to rise to 50 million deaths per annum by 2050^3^. Metal sequestering agents are increasingly recognised as unique therapeutic options since they restrict growth by depriving bacteria of metals essential for metabolism^4–6^. Such an approach resembles nutritional immunity in hindering pathogen access to vital metals, often by engaging sequestering proteins and other molecules^7–10^. For instance, calprotectin, a heterodimer composed of S100A8 and S100A9 proteins, is a significant factor in restricting the bioavailability of manganese and zinc^10–13^. Despite strategies to actively import essential metals from their surroundings^9,14^, evolution of resistance is challenging for microbes as multiple cellular targets are involved. Hence, these innate pathways have been retained as part of the effective defensive armoury of the immune response.

In Gram-negative bacteria, such as *Escherichia coli*, natural and synthetic metallophores are known to preferentially deprive cells of iron, manganese and zinc^15^. Some chelators, such as ethylenediaminetetraacetic acid (EDTA), can also disrupt outer membrane integrity, which is thought to be due to removal of stabilising metal ions^16–18^. In contrast, the antibacterial properties and cellular effects of small, metal-sequestering compounds against Gram-positive species has received considerably less attention. To help identify cellular targets of chelating agents and investigate how cells might adapt to metal deprivation, *Staphylococcus aureus*, an important human commensal and opportunistic drug-resistant pathogen^19,20^, was cultivated in the presence of low concentrations of either EDTA or diethylenetriamine pentamethylene phosphonic acid (DTPMP). EDTA is an aminocarboxylate hexadentate ligand, whereas DTPMP has a related nitrogenous core but with five pendant phosphonates replacing the carboxylates. Both have high affinities for metals, especially Fe^3+^ ^21,22^. These two chelators were selected because of their differential effects on *E. coli* cellular metal content^15^. EDTA primarily depletes *E. coli* of manganese with additional, lesser reductions in iron and zinc, while DTPMP deprives of iron^15^. This current study complements one carried out with *E. coli*, which isolated resistant strains with upregulated zinc and iron homoeostatic mechanisms^23^. Rather than metal uptake systems, in *S. aureus* the mutations were found to affect cell wall biosynthesis. Furthermore, EDTA and DTPMP behaved similarly in their effects against *S. aureus*, depriving cells of manganese with only modest reductions in iron and zinc levels. Significant changes in cell wall metabolism affecting peptidoglycan polymerisation and teichoic acid modification proved responsible for resistance by influencing the availability of surface-associated metals. The results reveal important details concerning the antibacterial action of chelators against Gram-positive bacteria with implications for the development of metal sequestering agents in the treatment of infection.

## Results

### Isolation of chelator-resistant mutants of *S. aureus*

**Fig. 1:**
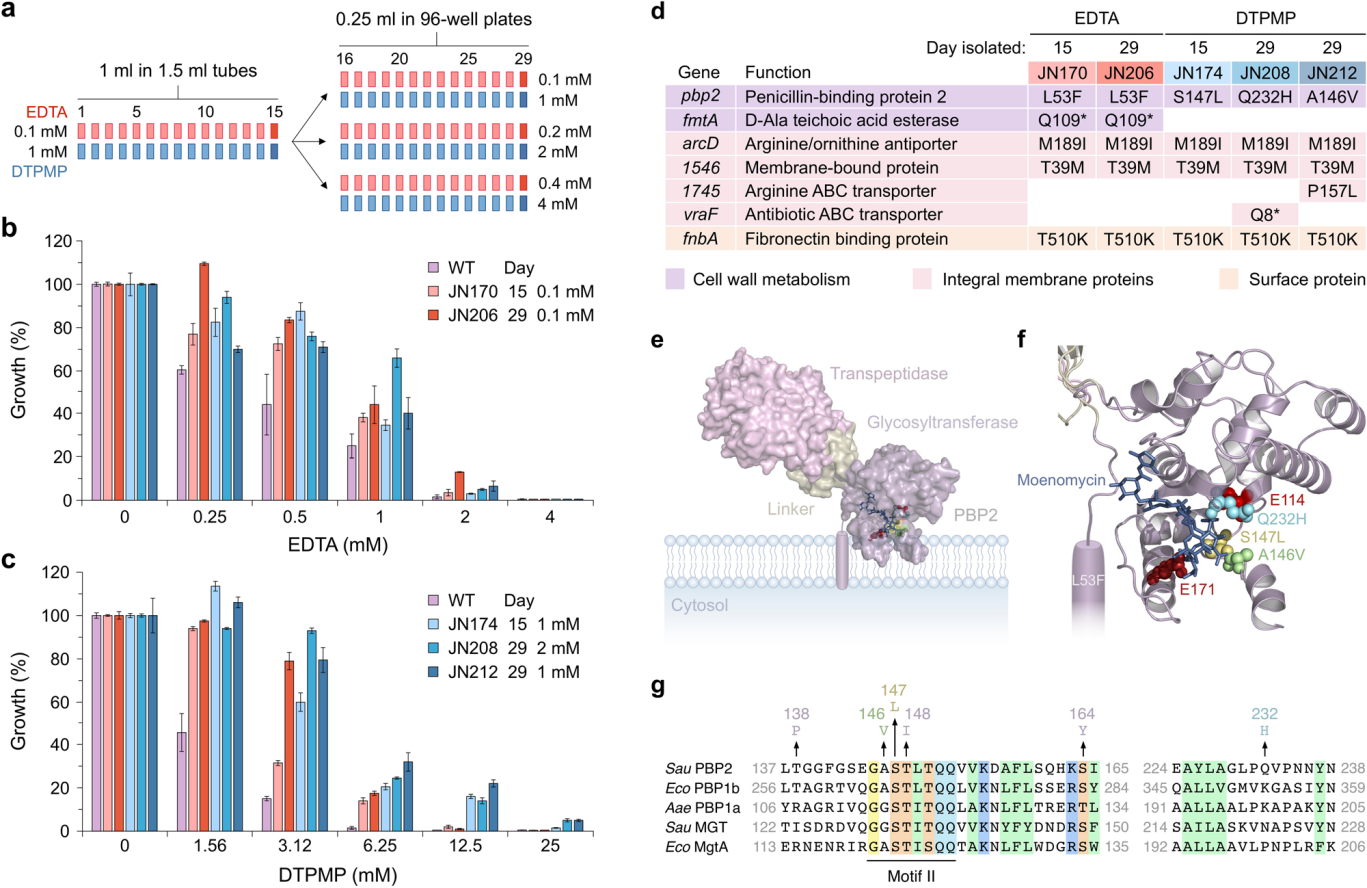
Isolation and characterisation of *S. aureus* chelator-resistant mutants. **a** Mutants resistant to either EDTA or DTPMP were obtained by repeated subculture for 15 and 29 days at sub-MIC levels of each chelant (see “Methods” and Table S1). **b**, **c** Growth in the presence of each chelator was measured at OD_600 nm_ and normalised against controls without chelant. Results represent the mean and standard deviation of an experiment performed in triplicate. An additional biological repeat produced similar results (Fig. S1a, b). The key in each panel shows the respective mutants isolated against EDTA (shades of red) and DTPMP (shades of blue), including the concentrations used for selection. The WT and all five chelant-selected strains were tested for resistance to EDTA (**b**) and DTPMP (**c**). **d** Key mutations identified by whole genome sequencing. Genes affected are grouped according to function and specific mutations listed for each strain. Missense mutations that change amino acid sequences of the gene product are shown and replacement of an amino acid with a stop codon is indicated by an asterisk. **e** Location of substitution mutations in the PBP2 glycosyltransferase domain. Structure of *S. aureus* PBP2 complexed with moenomycin (PDB: 2OLV) and its position in the bacterial cytoplasmic membrane. The transpeptidase, glycosyltransferase and intervening linker domains are highlighted. A single transmembrane helix tethers PBP2 to the membrane. **f** Location of PBP2 glycosyltransferase active site residues and mutations identified in this study. Active site glutamates (E114 and E171) are coloured red. Three mutations (A146V, S147L, Q232H) found in DTPMP isolates cluster close to the active site where the sugar backbone of peptidoglycan is assembled. The Glc*N*Ac-Mur*N*Ac substrate analogue moenomycin is represented in stick format. A fourth mutation, present in EDTA-isolated mutants (L53F), is located in the transmembrane helix that spans the lipid bilayer. In the AlphaFold model of PBP2 (AF-Q53729-F1) Leu-53 protrudes outwards from the alpha helix into the lipid-rich core of the bilayer. Structures were visualised in Pymol. **g** Sequence alignment in the vicinity of GT_51_ conserved motifs II and V in selected bacterial glycosyltransferases highlighting the location of A146V, S147L and Q232H mutations^33,34,58^. Selected additional mutations in the PBP2 glycosyltransferase domain isolated as suppressors of salt sensitivity in a Δ*0957* strain^41^ are also shown (S1: S164Y, S2: T138P and S4: T148I); an additional suppressor in PBP2 (S10) is identical to the S147L substitution. *S. aureus* (*Sau*), *E. coli* (*Eco*) and *Aquifex aeolicus* (*Aae*). Conserved residues present in all sequences are highlighted and GT_51_ motif II is labelled. Residue positions within each of the protein sequences is indicated and accession numbers are: PBP2 (F4NA87), PBP1b (P02919), PBP1a (O66874), MGT (Q93Q23) and MgtA (P46022).

*S. aureus* FDA209P, an MSSA strain^24^, was exposed to low concentrations of EDTA or DTPMP using the same experimental evolution strategy undertaken previously with *E. coli*^23^. After 15 and 29 days of daily sub-culture, with the same or higher concentrations of each chelator, single colonies were isolated (Fig. 1a), and five strains assayed for acquired resistance to EDTA and DTPMP (Fig. 1b, c). The two strains selected against EDTA displayed improved growth in the presence of this chelator relative to the parent strain (Figs. 1b and S1a). Similarly, three strains isolated at sub-MIC (minimum inhibitory concentration) levels of DTPMP proved more resistant to DTPMP than the WT (Figs. 1c and S1b). Unexpectedly, strains selected against EDTA showed improved resistance to DTPMP, and correspondingly, DTPMP-resistant strains also showed enhanced resistance to EDTA (Fig. 1b, c), implying a commonality in chelator mode of action and resistance mechanism. There were no major differences between the WT and the five mutants in colony morphology, although DTPMP-isolated strains JN174 and JN212 produced smaller colonies after overnight incubation (Fig. S2a–f). In liquid culture, in the absence of chelating agents, one of the EDTA mutants, JN206, showed an extended lag phase and slower exponential phase but ultimately reached a similar optical density as the WT (Fig. S2g). One of the DTPMP-isolated strains, JN212, displayed a reduced growth rate, failing to achieve the same optical density as the parent strain after 12 h incubation (Fig. S2g).

### Whole genome sequencing and identification of mutations in resistant strains

To identify mutations responsible for improved resistance of *S. aureus* to EDTA and DTPMP, the whole genome sequence of each strain, including the WT, was determined using an Illumina MiSeq platform^23^. A pipeline^25^ was employed to annotate genomic variants between the parent and derivative strains (Figs. 1d and S3). Key mutations affecting genes in all the chelator-resistant strains could be responsible for the changes that result in resistance (Fig. 1d). Rather than the anticipated metal uptake systems, the major changes affected cell surface molecules, membrane-bound channels and cell wall biosynthetic machinery. Specifically, these included single amino acid substitutions in PBP2 (Penicillin-binding protein 2), ArcD (an arginine:ornithine antiporter), SAFDA_1546 (an uncharacterised membrane protein) and FnbA (fibronectin-binding protein). Mutations affecting *arcD*, *SAFDA_1546* and *fnbA* are identical and may have arisen at the outset of the experiment, explaining why they are present in all strains. However, since they all affect integral membrane proteins and could beneficially alter cell surface charge or influence nutrient uptake, they could make some contribution to chelant resistance. Additional mutations considered to be significant included stop codons disrupting *fmtA*, which encodes a teichoic acid D-Alanine (D-Ala) esterase^26,27^ present in both EDTA-isolated strains, and *vraF* encoding part of the ABC transporter VraFG^28^ found in JN208, a strain selected against DTPMP (Fig. 1d). Importantly, VraF is a component of the GraXRS-VraFG complex, known to regulate the *dltABCD* operon responsible for D-alanylation of teichoic acids^28–30^. A substitution mutation in a putative arginine ABC transporter, *1745*, was unique to JN212. Additional mutations present in chelator-resistant strains were considered less likely to influence resistance because they are only present in a single strain, affect regions of a gene product that are poorly conserved, or are otherwise unlikely to be critical for the observed phenotype (Fig. S3). Subsequent experiments focussed on the involvement of *pbp2*, *fmtA* and *vraF* in chelator resistance as all directly influence either peptidoglycan assembly or teichoic acid modification.

### Reduced peptidoglycan cross-linking and increased cell wall thickness in mutants

All chelator-resistant strains carry mutations in PBP2 (Fig. 1d). Penicillin binding proteins (PBPs) are required for the synthesis and maintenance of peptidoglycan and *S. aureus* strain FDA209P has four in total^31,32^. PBP2 is singular in *S. aureus* in catalysing both glycosyltransferase (GTase), the polymerisation of glycan strands from lipid II precursors, and transpeptidation, the formation of peptide cross-links between these chains^32^. The presence of four distinct substitution mutations in PBP2 in each of the five chelator-resistant strains strongly suggested that they might fulfil an important role in EDTA and DTPMP resistance. Three of these mutations (A146V, S147L and Q232H), found only in the DTPMP isolates, cluster near the GTase active site E114 and E171 residues of PBP2 (Fig. 1e–g) in a channel where N-acetylglucosamine (Glc*N*Ac) and N-acetylmuramic acid (Mur*N*Ac) are fused to assemble the glycan chain^33^. Notably, PBP2 S147 is the equivalent of *S. aureus* MGT S132, responsible for binding Glc*N*Ac of lipid II through a backbone amide group^34^. EDTA-selected strains possess a different PBP2 mutation (L53F) in the transmembrane helix that spans the lipid bilayer and positions the enzyme for peptidoglycan synthesis^33^ (Fig. 1e, f). Substitution of leucine for phenylalanine may alter the conformation of the helix within the membrane, potentially affecting the anchoring position or dynamics of the embedded portion of the protein.

**Fig. 2:**
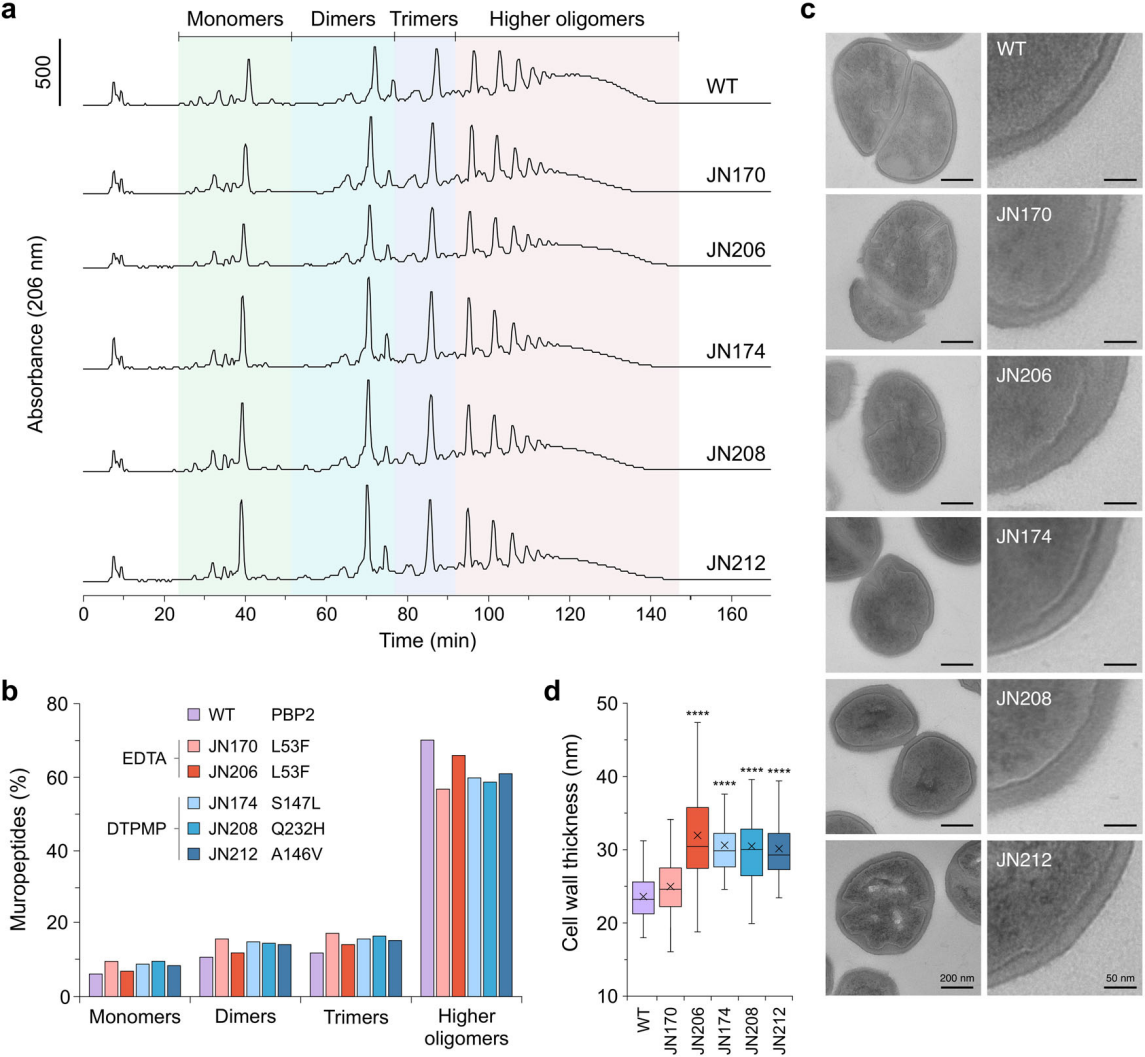
Reduced peptidoglycan cross-linking and increased cell wall thickness in mutant isolates. **a** Chelator-selected strains were cultivated, the peptidoglycan purified and the extent of cross-linking was determined by analysis of the muropeptide composition by HPLC. A representative data set is shown. **b** Relative proportion of monomer, dimer, trimers and higher muropeptide oligomers from the WT and mutants determined from the data in (**a**). An additional biological repeat produced similar results (Fig. S4). **c** TEM images of WT and mutant strains. Representative images are shown. **d** Measurement of cell wall thickness based on 100 cells, with average measurements taken at the four quadrant axes in each cell. Horizontal lines define the mean, x the median, boxes define the 25th percentiles, and whiskers the maximum and minimum values. One-way ANOVA analysis with a post hoc Dunnett test was used to compare the WT and mutants, ****p < 0.0001.

One of the characteristics of staphylococcal peptidoglycan is its high degree of cross-linking, with up to 90% of its muropeptides linked to adjacent glycan chains in the peptidoglycan mesh^35^. Since the chelator-resistant isolates all carry mutations in PBP2, the degree of peptidoglycan cross-linking was examined in comparison with the parental WT (Figs. 2 and S4). A reduction in cross-linking relative to the WT was apparent with all five mutants (Fig. 2a). The ‘hump’ in higher oligomers with the WT is reduced in the chelator-resistant strains and these mutants showed a corresponding increase in the proportion of monomer, dimer and trimer species (Fig. 2a, b). The three DTPMP strains (JN174, JN208 and JN212) showed a consistently higher percentage of monomer and dimer peaks than the WT or EDTA-selected mutants (Figs. 2a and S4a). It is noteworthy that EDTA-treated isolates, which both carry an identical L53F substitution in the transmembrane helix of PBP2, exhibited the same defect as the DTPMP mutants (Figs. 2a and S4a).

Given the observed changes in levels of peptidoglycan cross-linking, the same mutants were examined by transmission electron microscopy (TEM) to visualise any alterations in cell wall organisation. Representative images of sectioned whole cells and details of the cell wall are shown (Fig. 2c). Cells from the WT strain had an average wall thickness of 23.55 ± 2.98 nm, in line with previously published data ^36,37^; over 85% of the WT cell wall measurements were 20–30 nm and none exceeded 35 nm (Fig. 2d). Although, JN170 displayed a similar cell wall thickness as the WT, with an average of 24.92 ± 4.03 nm, it did exhibit more variability and a greater proportion of cells exceeding 30 nm. The remaining four mutant strains all possessed substantially thicker cell walls than the WT, ranging from 30 to 32 nm on average (Fig. 2c, d). JN206 manifested the thickest cell wall layer, with 14% of cells exceeding 40 nm. A small proportion of cells from the JN206 and JN208 samples had cell walls with a thickness >50 nm and exhibited extreme heterogeneity. In addition to the changes in cell wall dimensions, defects in cell division were apparent at a greater frequency in the mutant strains than the parental WT (Fig. 2c). The most frequent phenotype in the mutants was irregular septum formation, an increase in the presence of mesosomes and numerous cells with aberrant proportions (Fig. 2c). A fraction of the dividing cells also displayed extremely thick, heterogenous cell walls at the septum or in emergent cross walls.

### Influence of PBP2 mutations on susceptibility to moenomycin and osmotic stress

**Fig. 3:**
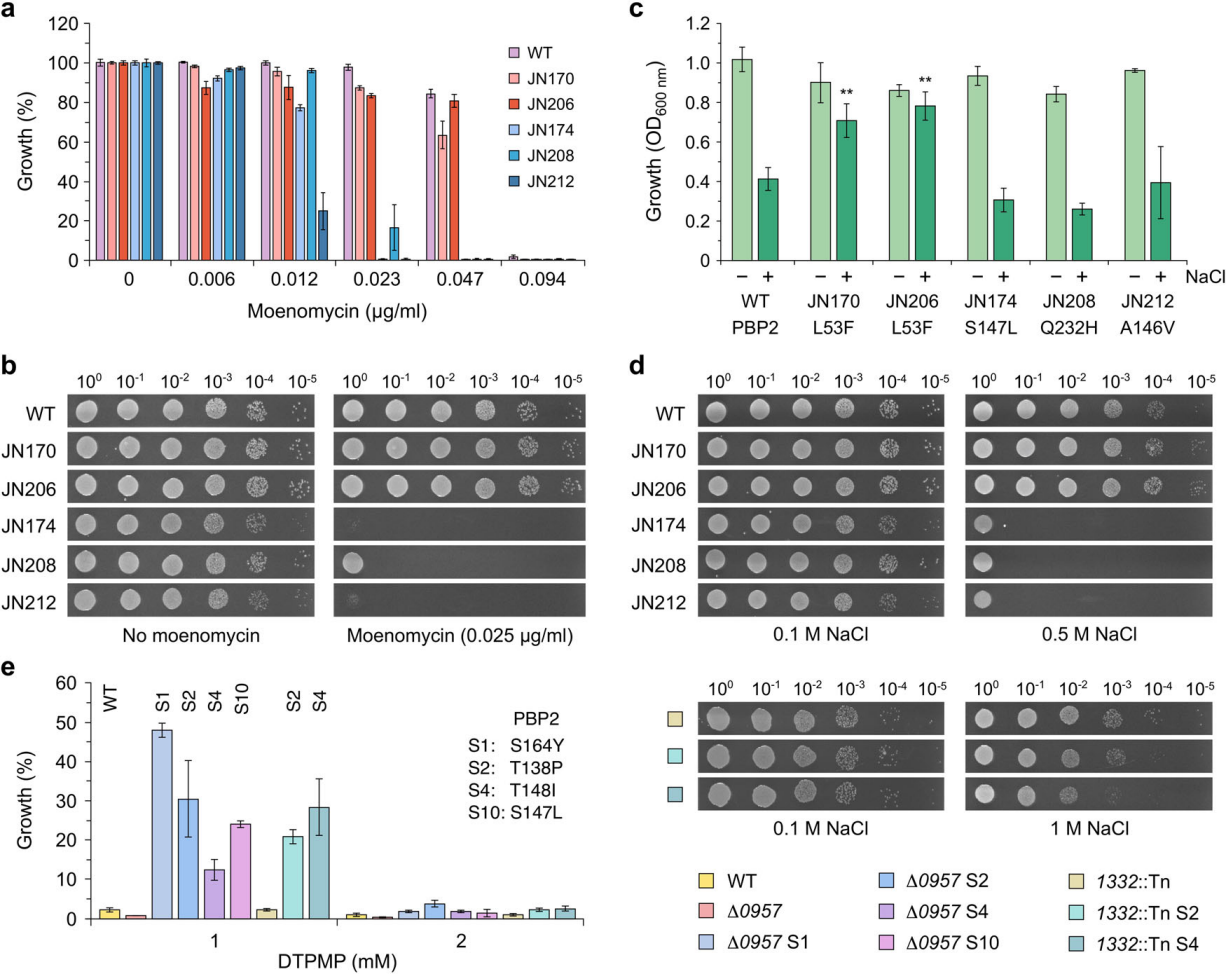
Influence of PBP2 mutations on sensitivity to moenomycin and osmotic stress. **a** Growth in the presence of moenomycin was measured at OD_600 nm_ and normalised against controls without antibiotic to give the percentage growth. Results represent the mean and standard deviation of an independent experiment performed in triplicate. An additional biological repeat produced similar results (Fig. S5). **b** Strains were grown to an OD_600 nm_ of 0.4, serial 10-fold dilutions performed and 10 µl volumes applied to the surface of LB agar plates in the presence or absence of moenomycin. The experiment was performed three times and one representative image is shown. **c** Salt resistance of mutants. Bacteria were cultivated in media containing either 0.1 M (–) or 0.5 M (+) NaCl and growth monitored. Results show growth after 6 h at 37 °C in LB (*n* = 3) and full growth curves are shown in Fig. S6). One-way ANOVA analysis with a post hoc Dunnett test was used to compare mutant strains to the WT, each in the presence of 0.5 M salt (**p < 0.01). **d** Susceptibility of mutants to high salt. Strains were grown and applied to the surface of LB agar plates (as in b) containing 0.1 or 0.5 M NaCl. The slow-growth phenotype of JN174 and JN212 is apparent here in samples without moenomycin and at 0.1 M salt. The experiment was performed three times and one representative image is shown. **e** Susceptibility of *S. aureus* USA300 PBP2 mutants isolated as suppressors of Δ*0957* to DTPMP and osmotic stress. Growth was measured in the presence of DTPMP and normalised against controls without chelant to give the percentage growth. Results represent the mean and standard deviation of an independent experiment performed in triplicate; appropriate parental controls were compared with strains carrying the different suppressor (S) mutations. An additional two biological repeats produced similar results (Fig. S7). Sensitivity of selected *S. aureus* USA300 PBP2 suppressor mutants to high salt (right). Strains with a *1332*::*Tn* insertion, as indicated by colour-coded boxes, were applied to the surface of LB agar plates containing 0.1 or 1 M NaCl.

Specific mutations in the glycosyltransferase active site of PBP2 confer *S. aureus* resistance to moenomycin by reducing antibiotic binding^38^; notably one of these, P234Q, is close to the Q232H substitution identified here (Fig. 1g). Due to the nature of its target^39^ and the potential significance of the glycosyltransferase mutations present in PBP2 (Fig. 1d), moenomycin was chosen to probe differences in cell wall synthesis between isolates. The EDTA-selected strains displayed a similar sensitivity profile as the WT in liquid culture (Figs. 3a and S5) and to moenomycin incorporated in agar plates (Fig. 3b). In contrast, the DTPMP-selected isolates had increased susceptibility to this antibiotic (Figs. 3a and S5), especially on agar plates (Fig. 3b). One of the DTPMP-isolated mutants, JN208, showed slightly better resistance to moenomycin relative to the other two strains (Figs. 3a, b and S5).

The cell wall of *S. aureus* is essential for maintaining cell integrity against osmotic pressure, ensuring the preservation of appropriate concentrations of solutes and preventing cell lysis^40^. *S. aureus* has a relatively high salt tolerance relative to other bacterial species, even Gram-negatives, mediated through its ability to increase turgor, initially through elevation of intracellular osmolytes, such as potassium and glycine betaine^40^. Tolerance also depends on cell wall integrity and alterations in peptidoglycan and teichoic acid polymers have been linked to the capacity of a community-acquired methicillin-resistant *S. aureus* USA300 strain to withstand osmotic stress. A study by Schuster et al.^41^ identified a gene (*SAUSA300_0957*) important for sodium chloride resistance, which when deleted increased peptidoglycan cross-linking. Significantly, the salt sensitivity of this mutant could be suppressed by amino acid substitutions in the glycosyltransferase domain of PBP2, restoring peptidoglycan cross-linking to the same level as the WT^41^. One of the Δ*975* suppressor mutations in PBP2 was S147L, identical to that found in the DTPMP-resistant strain, JN174 (Fig. 1d) and other suppressor mutations are located nearby (Fig. 1g). Like the Δ*975* suppressed strain^41^, JN174 exhibited a reduction in peptidoglycan cross-linking (Fig. 2).

The links between PBP2, peptidoglycan cross-linking and osmotic stress, prompted us to assess the chelator-resistant mutants for their capacity to grow in the presence of high salt (Fig. 3c, d). Bacteria cultured in standard Luria-Bertani (LB; 0.1 M NaCl) and high salt LB broth (0.5 M NaCl) were examined (Figs. 3c andS6). The WT showed significantly impaired growth in high salt medium (Fig. 3c) and all three of the DTPMP-isolated strains displayed a similar level of sensitivity (Figs. 3c and S6). In contrast, the EDTA-selected mutants were much more resistant to osmotic stress, growing almost as well in LB containing 0.5 M NaCl as the standard LB (Figs. 3c and S6). The susceptibility of strains to high salt was also investigated using LB agar plates containing 0.1 and 0.5 M NaCl (Fig. 3d). A slight improvement in growth of the EDTA-isolated mutants relative to the wild-type in high salt conditions was noted (Fig. 3d; compare growth at the 10^−5^ dilution), although it was less obvious than that seen in LB broth (Figs. 3c and S6). In contrast, the DTPMP-isolated strains exhibited an extreme salt sensitivity (Fig. 3d), which was not apparent in liquid culture (Fig. 3c).

Since *S. aureus* Δ*957* strains carrying mutations in PBP2 reduce peptidoglycan cross-linking^41^, four of the USA300 suppressor strains were checked to see if they could promote resistance to DTPMP. All four PBP2 mutations showed improved growth in the presence of DTPMP relative to their Δ*957* parent (Figs. 3e and S7). Two of the suppressor mutants (PBP2 T138P and T148I) in a *957*^*+*^ background with insertions in an unrelated gene (*1332*::*Tn*) with no role in cell wall metabolism^41^ also showed improved growth in the presence of DTPMP (Figs. 3e and S7). These two suppressors were also assessed for their susceptibility to high osmotic stress (Fig. 3e). *S. aureus* USA300 is more resistant to high salt conditions than the FDA209P strain (Fig. 3d). However, the two strains carrying mutations in *pbp2* did show reduced growth relative to their *pbp2*^+^ parent (Fig. 3e), in keeping with reduced osmotic stress tolerance due to decreased peptidoglycan cross-linking.

### Altered mutant susceptibility to cell-wall targeting agents and changes in surface charge

**Fig. 4:**
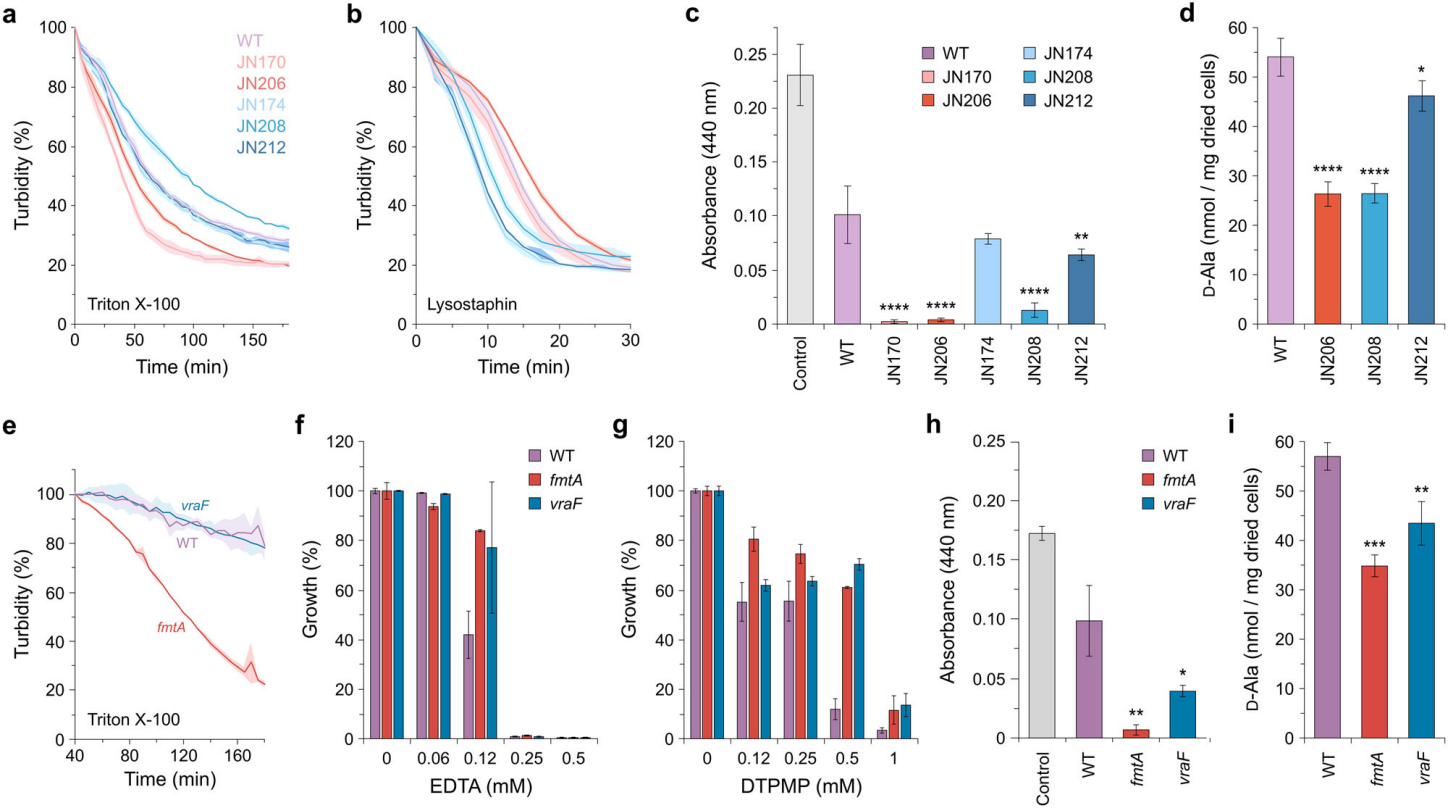
Susceptibility of *S. aureus* mutants to cell wall agents and alterations in cell surface charge. **a**, **e** Sensitivity of *S. aureus* mutants to Triton X-100. Triton X-100 (0.1–1%) was added to PBS-washed cell suspensions and turbidity monitored at OD_600 nm_. Results represent the mean (*n* = 3) with standard deviation indicated by shading. **b** Sensitivity of *S. aureus* mutants to lysostaphin. Lysostaphin (2.5 µg/ml) was added to PBS-washed cell suspensions and turbidity monitored at OD_600 nm_. Results represent the mean (*n* = 3) with standard deviation indicated by shading. **c**, **h** Changes in bacterial surface charge in a cytochrome *c* binding assay. Cytochrome *c* (5 mg/ml) was added to concentrated cells and the supernatant following centrifugation to pellet cells measured at A_440 nm_ (*n* = 3). **d**, **i** D-Ala content of *S. aureus* cells determined by HPLC analysis. Results represent the mean (*n* = 3) with standard deviation. **f**, **g** Susceptibility of *S. aureus fmtA* and *vraF* mutants to EDTA and DTPMP. Growth in the presence of each chelator was measured at OD_600 nm_ and normalised against controls without treatment to give the percentage growth. Results represent the mean and standard deviation of an independent experiment performed in triplicate. An additional biological repeat of each treatment produced similar results (Fig. S11a,b). One-way ANOVA analysis with a post hoc Dunnett test was used to compare mutant strains to the WT; *p < 0.05, **p < 0.01, ***p < 0.001 and ****p < 0.0001.

Changes in envelope architecture in the *S. aureus* mutants were investigated further by exposure to cell wall-damaging agents to clarify changes that might be linked to chelator resistance. Sensitivity to the non-ionic detergent Triton X-100 was assessed first. Triton X-100 specifically stimulates the release of acylated lipoteichoic acids (LTAs) in *S. aureus*, inducing bacterial autolysis as a consequence of reduced cross-linking and digestion of peptidoglycan^42^. Cells were grown, exposed to the detergent and turbidity monitored over time. The WT gradually lost turbidity over several hours and the DTPMP resistant strains followed a similar trend (Fig. 4a), although JN208 displayed a modest increased resistance. In contrast, both EDTA-resistant mutants (JN170 and JN206) showed increased susceptibility indicating modifications that render the cell wall more accessible to this surfactant.

A similar assay was conducted to assess susceptibility to the peptidoglycan endopeptidase lysostaphin^43^. In contrast to detergent-exposed cells, it was the DTPMP-isolated mutants that displayed increased sensitivity to lysostaphin (Fig. 4b). The EDTA-isolated mutants behaved much like the WT, although a slight improvement in resistance was evident at later timepoints with JN206 (Fig. 4b). The results with Triton X-100 and lysostaphin again highlight significant differences between the EDTA and DTPMP isolates, despite having developed dual chelator resistance.

Mutations affecting the composition and structure of peptidoglycan and teichoic acids in the *S. aureus* chelator-isolated strains could alter the net surface charge. The binding of cells to cytochrome *c* was evaluated to investigate this possibility. Cytochrome *c* is a small, mitochondrial haemoprotein with a net positive charge and distinctive red colour^44^. WT and mutant cells were mixed with cytochrome *c*, subjected to centrifugation and the supernatant assayed for the presence of pigment. The WT cells removed approximately half of the cytochrome *c* from solution in keeping with their overall negative outer surface charge (Fig. 4c). Significantly, almost all the cytochrome *c* was removed from solution when mixed with either of the EDTA-selected mutants (Fig. 4c). The results suggest that these strains, JN170 and JN206, have a much more negatively charged cell surface than the WT. A similar effect, if not quite so marked, was noted with two of the DTPMP-isolated mutants, JN208 and JN212 (Fig. 4c). The remaining DTPMP strain (JN174) behaved much like the WT parent (Fig. 4c).

Populating polyanionic teichoic acids with D-Ala reduces negative cell surface charge in *S. aureus* and the capacity to modulate D-Ala content is critical for resistance to cationic antibacterial agents and infection^29,45^. The extent of teichoic acid D-alanylation in WT and mutant cells was therefore probed by the release of D-Ala under alkaline conditions, Marfey’s reagent derivatization, followed by detection (Fig. S8a)^46^. JN206 and JN208 contained considerably less (~2-fold) D-Ala than the WT, whereas JN212 displayed only a small decrease relative to WT cells (Fig. 4d).

### Contribution of *fmtA* and *vraF* null mutants to the observed phenotypes

In addition to a missense mutation in *pbp2*, both EDTA-selected strains carry a nonsense mutation in *fmtA* that eliminates more than two-thirds of the protein (Fig. 1d), including the esterase catalytic site^26^. FmtA hydrolyses the ester bond between D-Ala and the teichoic acid backbone, thereby modulating cell surface charge according to physiological requirements^27^. One of the DTPMP-selected strains, JN208, carries a stop codon at Gln-8 of *vraF* (Fig. 1d). *S. aureus vraF* mutants are susceptible to polymyxin B (ref. ^47^; Fig. S9a) and only JN208 of the chelator-selected strains showed an equivalent sensitivity to this antibiotic (Fig. S9b), confirming that this isolate is indeed deficient in VraF. The VraFG ABC transporter participates in the export of cationic antimicrobial peptides, antibiotics and bacteriocins and helps regulate the two-component GraXRS sensory network, which includes the *dltABCD* operon involved in D-alanylation of teichoic acids^48^. Hence the absence of either *fmtA* or *vraF* products could be responsible for several of the phenotypes observed in the relevant chelator-resistant strains. To investigate their contribution further, *S. aureus* USA300 *fmtA*::*emr* and *vraF*::*emr* transposon insertion mutants were obtained from the Nebraska Transposon Mutant Library^49^.

*S. aureus fmtA* mutants are sensitive to Triton X-100 ^50–52^ and the *fmtA* mutant, but not the *vraF* strain, proved more susceptible to detergent exposure (Fig. 4e). Neither mutant was more sensitive than the WT to lysostaphin (Fig. S10a), also true of strains carrying mutations in *pbp2* (Fig. S10b). Confirming their direct involvement in chelator resistance, both the *fmtA* and *vraF* mutant strains showed improved growth relative to the WT at several concentrations of EDTA (Figs. 4f and S11a) and DTPMP (Figs. 4g and S11b). The *fmtA* mutant showed the same sensitivity to moenomycin as the WT, while the *vraF* mutant strain yielded a modest improvement in relative growth at a single concentration of the antibiotic (Fig. S11c). When moenomycin was incorporated in agar plates, both *fmtA* and *vraF* mutants displayed better growth than the WT (Fig. S11d), whereas no change in resistance to osmotic stress was evident (Fig. S11e). Changes to cell surface charge were investigated using the cytochrome *c* binding assay (Fig. 4h). As before with the FDA209P strain (Fig. 4c), the *S. aureus* USA300 WT removed about half of the cytochrome *c* from solution (Fig. 4h). Greater amounts of the haemoprotein were bound by the USA300 cells lacking *fmtA* and *vraF*, especially with the former (Fig. 4h). In keeping with an increased cell surface negative charge, the *fmtA* and *vraF* mutants contained reduced quantities of teichoic acid-associated D-Ala (Fig. 4i). The results reveal a clear correlation between teichoic acid D-Ala depletion, cell surface negative charge and chelator resistance when FmtA and VraF activities are unavailable (Fig. 4f, g). Strains with mutations in the PBP2 glycosyltransferase domain also showed indications of an increased negative charge in cytochrome *c* assays (Fig. S10c).

### Metal content of chelator-resistant mutants

To understand how the modified bacterial envelope in chelator-selected strains might improve resistance to metal restriction, the cellular concentration of six transition metals was probed by inductively coupled plasma mass spectrometry (ICP-MS). The WT and two representative mutant strains (based on mutations in either *fmtA* or *vraF* and phenotypic differences), JN206 and JN208, were exposed to concentrations of each chelator that inhibited the growth of mid-log bacteria by ~10–30% (Fig. S12). As noted in smaller scale cultures (Fig. 1b, c), improved growth relative to the WT was evident with both mutants in the presence of either EDTA (Fig. S12a) or DTPMP (Fig. S12b).

**Fig. 5:**
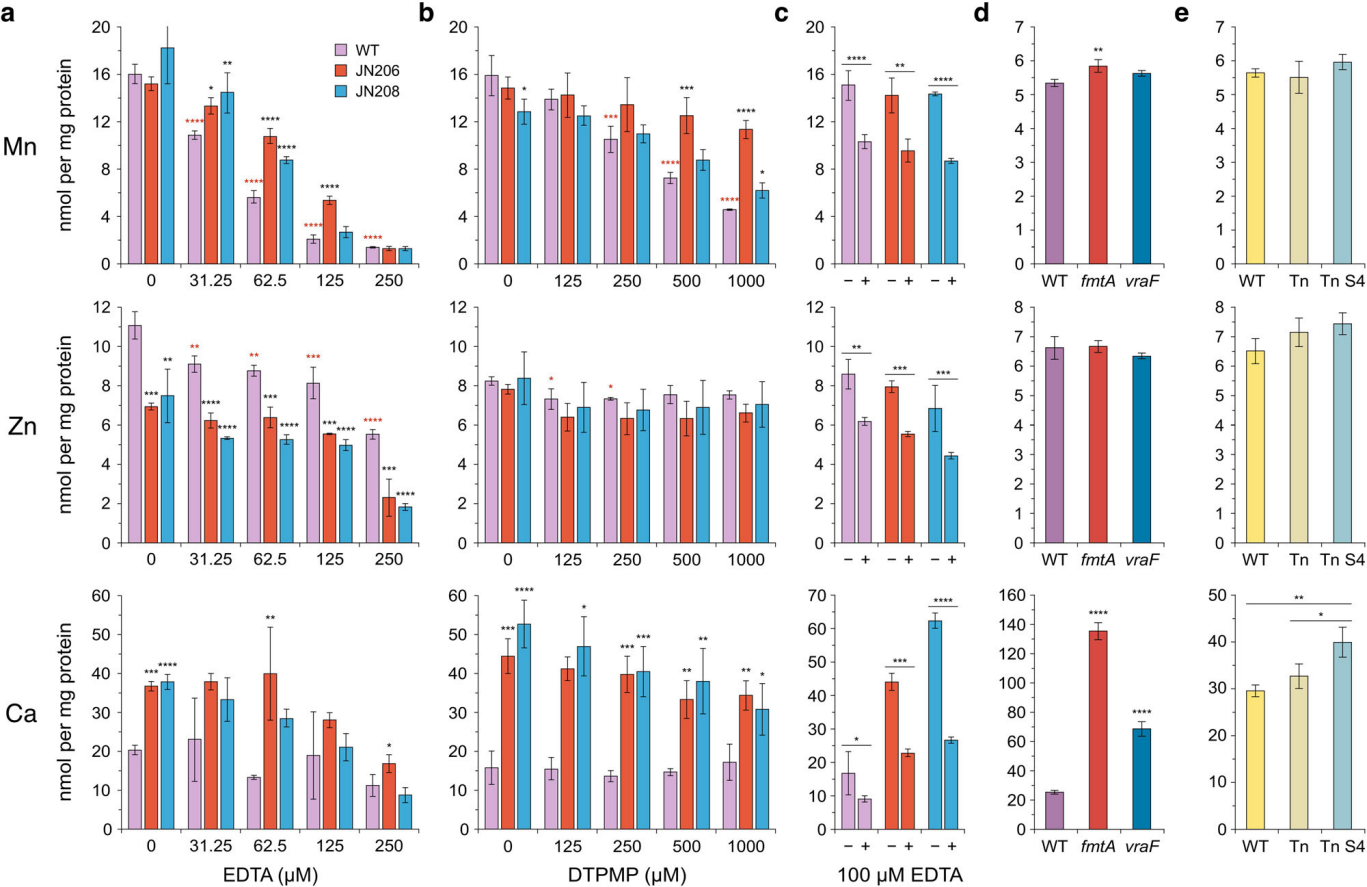
Effect of chelators on the cellular Mn, Zn and Ca composition of *S. aureus* mutants. **a**, **b** Bacteria (50 ml) were cultured to early log-phase in the presence of EDTA or DTPMP producing growth inhibition of 10–30% and untreated controls set up in parallel. Amounts of each metal were determined by ICP-MS and presented in nmol per mg of total cellular proteins. **c** Comparisons were made between cells with or without EDTA in the wash buffer to distinguish surface-associated and cytosolic metals. **d** Comparison of cellular metal content between the USA300 WT and *fmtA* and *vraF* mutant strains. **e** Total cellular metal content USA300 WT and mutants carrying a *1332*::*Tn* insertion (Tn) and the *1332*::*Tn* insertion combined with the *pbp2* T148I mutation (Tn S4). One-way ANOVA analysis with post hoc Dunnett test (**a**, **b**, **d**) or Tukey test (**e**) was used to compare each chelant concentration against the relevant control (*n* = 3). A Student’s *t* test was used to compare cells with or without EDTA in the wash buffer in (**c**) (*n* = 3). *p < 0.05, **p < 0.01, ***p < 0.001 and ****p < 0.0001. Asterisks in red (**a**, **b**) refer to comparisons between treated and untreated WT samples, those in black compare JN206 and JN208 samples against the WT under the same conditions.

The impact of these two metal sequestering agents on cellular metal levels has not been previously characterised in Gram-positive bacteria. Unlike *E. coli*^15^, EDTA and DTPMP had similar effects on *S. aureus* metal homoeostasis, with the greatest reductions affecting manganese (Fig. 5a, b). A modest reduction in iron levels was also noted in the WT, but not at higher chelator concentrations where growth inhibition was greatest (Fig. S13a, b). Zinc levels were reduced slightly by EDTA, whereas calcium, magnesium and copper levels remained stable in response to treatment (Figs. 5 and S13). The similarities in targeted metals explain why the mutants exhibit resistance to both chelators (Fig. 1b, c).

The two chelator-selected strains were examined next to see whether their ability to resist EDTA and DTPMP was due to changes in their metal handling capabilities. In the absence of chelator, it was notable that the cellular concentration of calcium was substantially greater (4–5-fold) in JN206 and JN208 over the WT (Figs. 5a, b and S14). JN206, but not WT or JN208, accumulated iron at increased cell density in the absence of chelator (Figs. S13b and S14), while JN208 had lower cellular copper and magnesium (Fig. S14). Both mutant strains maintained elevated calcium levels at all concentrations of DTPMP (Fig. 5b) but less so with EDTA (Fig. 5a). JN206 and JN208, also retained proportionally higher levels of manganese than the WT when exposed to EDTA (Fig. 5a) or DTPMP (Fig. 5b). Iron levels in the two mutants were comparable with the WT at each chelant concentration (Fig. S13a, b). The lower zinc levels in JN206 and JN208 relative to the WT (Fig. 5a) were reduced further by EDTA (Fig. 5a) but were unaffected by DTPMP treatment (Fig. 5b).

Whole cell analysis of metals typically incorporates wash steps with an EDTA-containing buffer prior to ICP-MS analysis to remove extracellular and cell surface-associated metals^53^. To determine which metals might be more readily available at the cell surface, the three strains were cultivated as before in the absence of chelators, and half of the pelleted cells were processed in regular buffer while the remainder were washed in buffer supplemented with EDTA to remove cell surface-associated metals. Only 9–14% of the iron, magnesium and copper was lost following a single EDTA wash step in the WT (Fig. S13c). In contrast, levels of manganese, zinc and calcium in the WT were reduced by 26–30% (Fig. 5c), consistent with these three metal ions being more accessible at the cell surface. The two mutants showed similar levels of metal removal when EDTA was incorporated in the wash buffer, except for calcium where approximately twice as much (48–57%) was eliminated (Fig. 5c). Despite this reduction, the two chelator-resistant strains still retained 2.5–3-fold higher levels of calcium than the WT (Fig. 5c).

The cellular metal content of the DTPMP-selected strain JN212 was also examined by ICP-MS (Fig. S15a, c). Under the conditions used, no improved growth (Fig. S15b, d) was observed and the mutant behaved much like the WT following exposure to EDTA (Fig. S15a) and DTPMP (Fig. S15c). Cellular metal levels were generally much lower than the WT even in the absence of chelator, markedly so with zinc and magnesium. Elevated calcium was apparent in cells exposed to DTPMP (Fig. S15c) but not in those involving EDTA (Fig. S15a). Experimental variability and decreased metal content could be a consequence of the slow growth phenotype of this strain (Fig. S2) and the resulting lower cell density used for elemental analysis.

### Contribution of FmtA, VraF and PBP2 to metal availability

The cellular metal content of strains carrying single mutations in *fmtA, vraF* and *pbp2* was examined to clarify their contribution to changes in metal bioavailability in the EDTA and DTPMP-selected isolates. Strains carrying the *fmtA*::*emr* and *vraF*::*emr* alleles were compared with the relevant USA300 WT. Cells from both mutants contained substantially higher levels of calcium relative to the WT (Fig. 5d), especially with *fmtA*, consistent with the loss of function of these genes in JN206 and JN208, respectively (Fig. 5a, b). While iron levels were lower than the WT in both mutants (Fig. S13d), none of the other metals were substantially affected (Figs. 5d and S13d). One of the Δ*957* suppressor mutants, *pbp2* S4 (T148I) in a *1332*::*Tn* background, which is known to have reduced peptidoglycan cross-linking^41^, was also examined. Relative to its parent genotype (*1332*::*Tn*), the S4 allele showed a small, but significant, increase in calcium but no alteration in the concentration of any of the five other metals (Figs. 5e and S13e). Elevated basal calcium levels, therefore, coincide with mutation of any of these three genes and, at least for JN206 and JN208, are associated with the retention of cell surface-associated manganese.

### Chelator-selected mutants are not resistant to calprotectin

During infection, host calprotectin (CP) sequesters transitions metals, including manganese and zinc, to restrict the proliferation of invading pathogens^10–13^. Manganese, and to a lesser extent zinc, are also the metals preferentially depleted by exposure of *S. aureus* to EDTA or DTPMP (Fig. 5). The influence of CP on the growth of chelator-resistant strains was therefore investigated. Significantly, none of the mutants showed improved CP resistance relative to the WT, with all strains experiencing growth inhibition of ~70% at 200 µg/ml of CP (Fig. S16a). There was also no major difference in CP resistance between the USA300 WT, *fmtA* and *vraF* mutant strains (Fig. S16b), nor a strain with a T148I mutation in the PBP2 GT domain (Fig. S16c). These findings highlight differences in how CP and chelators accomplish metal sequestration, suggesting that *S. aureus* cell wall penetration is necessary for the antibacterial activity of EDTA and DTPMP.

## Discussion

**Fig. 6:**
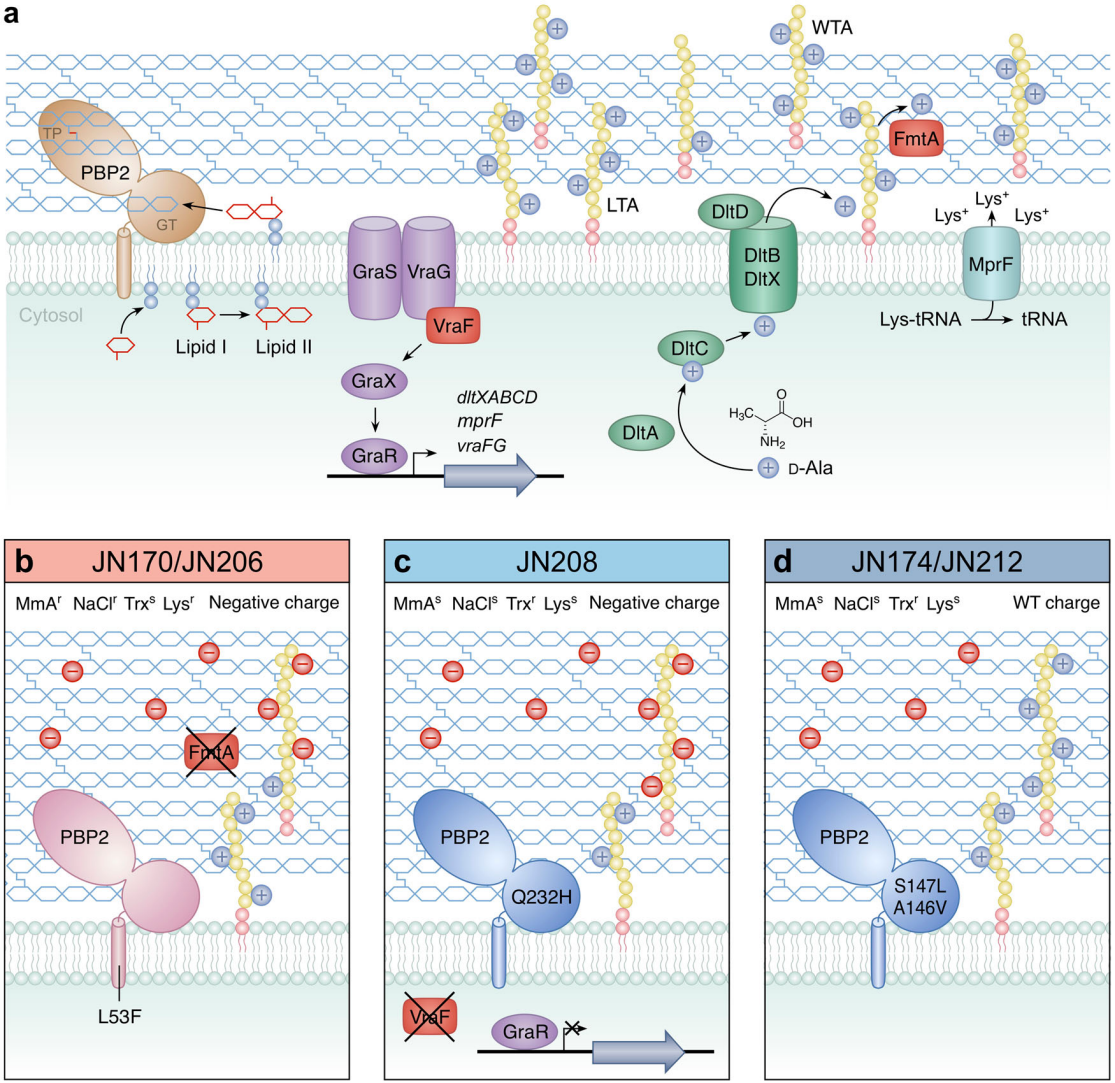
Components of the *S. aureus* cell wall machinery associated with resistance to EDTA and DTPMP. **a** Relevant peptidoglycan synthesis and teichoic acid modification proteins are illustrated. Peptidoglycan monomers, synthesised as lipid-linked precursors (lipid II), are transported across the cytoplasmic membrane prior to insertion into the sacculus network by PBPs^101^. PBP2 has both glycosyltransferase (GT) and transpeptidase (TP) activities required for elongation of glycan chains and formation of peptide bonds, respectively^102^. VraF, as part of a complex with VraG and alongside GraXRS, is involved in regulation of various genes that affect cell surface charge, most notably the *dltXABCD* operon whose products populate teichoic acids with D-Alanine (D-Ala), adding a greater positive charge (+). FmtA is an esterase that removes D-Ala from teichoic acid embedded in peptidoglycan and is implicated in removing positive charge. Lipoteichoic acids (LTA) and wall teichoic acids (WTA) are also indicated. **b**–**d** Effect of different mutations isolated in this study that promote EDTA and DTPMP resistance. All of the strains show a thicker cell wall, likely due to mutations in PBP2 that produce longer glycan chains with fewer cross-links. The two EDTA-selected strains (JN170/JN206) also lack *fmtA*. The three DTPMP strains have mutations affecting the GT domain of PBP2, while JN208 also has a null allele in *vraF*. Several of these strains display an increased negative (–) charge. Resistance (^r^) and sensitivity (^s^) to cell wall-targeting agents, moenomycin (MmA), osmotic stress (NaCl), Triton X-100 (Trx) and lysostaphin (Lys).

*S. aureus* cultivated in the presence of low levels of EDTA or DTPMP yielded variants with acquired resistance to each metal sequestering agent. Unexpectedly, strains selected against EDTA also showed improved resistance to DTPMP, and vice versa, implying a common mechanism that promotes resistance to both chelators. Whole genome sequencing identified mutations associated with changes in surface proteins, membrane transporters and cell wall processing (Fig. 6a), instead of the anticipated metal uptake systems. The most significant of these were *pbp2* substitution mutations present in all strains and null alleles in either *fmtA*, found in both EDTA strains, or *vraF*, in one of the three DTPMP isolates. A range of cell wall-targeting agents was employed to explore irregularities in the cell envelope of chelator-resistant strains and how these modifications enable resistance to metal deprivation. All isolates had acquired a substantially thickened cell wall and corresponding reduction in peptidoglycan cross-linking.

The EDTA-selected strains carry a PBP2 mutation in the transmembrane helix that secures the enzyme to the cytoplasmic membrane (Fig. 6b). This change could alter the positioning of the glycosyltransferase domain to reduce or increase glycan polymer synthesis activity, accounting for the reduced peptidoglycan cross-linking evident in these mutants. The membrane anchor of *Streptococcus pneumoniae* PBP2a is known to be critical for peptidoglycan chain length processivity^54^. Given that the mutants possess thicker cell walls, it is possible that the altered PBP2 generates longer chains or produces them at a faster rate, thus indirectly reducing the formation of peptide linkages. It has been shown for other bi-functional PBPs that both activities are coupled and GTase rate affects the TPase^55–57^. Cell wall functionality and integrity do not appear to be compromised since the EDTA-selected strains showed no increased susceptibility to the peptidoglycan synthesis inhibitor moenomycin or lysostaphin endopeptidase. However, the L53F substitution in PBP2 is probably responsible for the enhanced salt resistance of these mutants.

Each of the three DTPMP-selected mutants carries a unique substitution mutation in the GT domain of PBP2 and share broadly similar phenotypic responses. There were, however, differences between JN208 and the JN174/JN212 pair, some of which can be attributed to specific effects on GT functionality, in addition to the *vraF* mutation found only in JN208 (Fig. 6c). All DTPMP isolates exhibited greater susceptibility than the WT to moenomycin, osmotic stress, and lysostaphin. These phenotypes suggest a more accessible and structurally impaired sacculus, distinct from that found in the EDTA-selected variants. While the DTPMP mutants all have substitutions in the GT domain of PBP2, these affect different residues. JN174 and JN212 behaved similarly suggesting that the core mutations in PBP2, located alongside each other (S147A and A146V) in GT_51_ motif II^33,34,58^, are primarily responsible for the behaviour of these two strains (Fig. 6d). Experiments with USA300 strains carrying single mutations in the PBP2 GT domain support this conclusion^41^. Contrastingly, JN208 has a Q232H substitution mutation in PBP2 located adjacent to GT_51_ motif V. This mutated residue lies next to a conserved proline (P231) that resides within a cleft in PBP2 where the glycan chain is positioned during polymerisation^59^. Another surface-exposed proline (P234) when changed to glutamine produces moenomycin resistance and a reduced glycan chain length^38^. Examination of previously isolated GT domain substitution mutations^41^, verified a direct contribution of the PBP2 changes to DTPMP resistance.

Changes in cell surface charge were a major factor in chelator resistance and can be primarily attributed to the presence of additional mutations in *fmtA* and *vraF*. Mutation of either gene conferred improved EDTA and DTPMP resistance even in the absence of associated alterations to PBP2.

The *fmtA* mutation, found only in the EDTA isolates, introduces a stop codon at Gln109 in the 397-residue protein, eliminating the FmtA catalytic site^60^ and its capacity to liberate D-Ala residues from lipoteichoic acids (LTAs) attached to the outer leaflet of the cytoplasmic membrane and wall teichoic acids (WTAs) covalently linked to peptidoglycan (Fig. 6a)^27,61^. The net negative charge on both teichoic acid polyol-phosphate polymers is partially masked when D-Ala is added to the teichoic acids through DltXABCD esterification (Fig. 6a)^45^. FmtA balances D-Ala levels by hydrolysing the ester bond between amino acid and the teichoic acid backbone, modulating cell surface charge according to physiological requirements^50–52^. The Triton X-100 hypersensitivity of EDTA-selected strains is due to the loss of *fmtA* function as noted previously^50–52^. Reduced teichoic acid D-Ala content and an increased negative charge also coincided with inactivation of *fmtA*. Cells deficient in *fmtA* might be expected to have a more positive external charge due to an inability to eliminate D-Ala from teichoic acids. Indeed, elevated levels of D-Ala in purified WTAs have been reported in Δ*fmtA* mutants compared to *fmtA*^+^ strains^27^. These findings contradict those described here, although could be explained by changes in the extent of WTA over LTA D-alanylation or a decline in overall teichoic acid abundance in cells lacking the FmtA esterase^27^. The absence of D-Ala in LTA and WTA in *dlt* operon mutants of *S. aureus* and *Bacillus subtilis* is known to increase cell surface negative charge and susceptibility to cell wall-targeting antibiotics^44,62^. Sensitivity to acidic conditions in *fmtA* mutants^63^ also fits these features and may promote movement of ions, including metals, into the cell.

Changes in cell surface charge were also linked to the *vraF* mutation in the DTPMP-selected strain JN208 (Fig. 6c). The VraFG ABC transporter is implicated in the export of antibiotics, cationic antimicrobial peptides and bacteriocins and is regulated by the GraRS sensory system^48^. GraRS and VraFG are overexpressed in vancomycin-intermediate *S. aureus* (VISA) strains and mutations in *graR* and *vraG* confer hypersensitivity to vancomycin and polymyxin B^48^, confirmed here for the latter with JN208 and *vraF* deficient cells. VraF, as with FmtA, is associated with teichoic acid D-alanylation, notably by regulating expression of the *dltXABCD* operon as a component of the GraXSR-VraFG signalling system (Fig. 6a)^28,64^. An increased cell surface negative charge and lowered D-Ala content were apparent in strains lacking the *vraF* product. Mutations in *vraF* and the ensuing alterations in cell surface charge are frequently linked to antibiotic susceptibility in *S. aureus*. Increased negativity of the cell surface is associated with a greater susceptibility to cationic antimicrobial peptides^47^, daptomycin^65^ and vancomycin^48^. Conversely, an increased positive cell surface charge resulting from a point mutation in *vraF* (K84E) confers improved resistance to daptomycin and vancomycin^66^. Furthermore, depletion of LTA levels can mediate resistance to β-lactam antibiotics, a feature dependent upon the activities of VraFG and GraSR^67^. Reduction of LTAs in this context is achieved through mutation in *pgl*, a component of the pentose-phosphate pathway (PPP)^67^. The PPP is integrally linked with cell wall homoeostasis and, intriguingly, the enzyme ribose-phosphate pyrophosphokinase (Prs) involved in the latter stages of the PPP requires Mg(II) or Mn(II) for maximal activity^68^, and could therefore be a target for inhibition by chelators. In support of this, supplementation with copper ions induced PPP arrest in *S. aureus*, a likely consequence of Prs mismetallation^69^.

How are alterations in cell envelope architecture responsible for increased resistance to EDTA and DTPMP? Both chelators selectively depleted cellular manganese levels, an essential metal for *S. aureus*^13,70,71^. In addition, EDTA, but not DTPMP, reduced zinc levels. Those chelant-selected mutants with a greater negative charge, JN206 and JN208, missing *fmtA* and *vraF*, respectively, were found to contain substantially higher levels of calcium than WT cells. The thicker cell wall layer, rich in carboxylates combined with teichoic acid phosphates with reduced D-Ala content, appears to provide an expanded reservoir for calcium that more effectively protects against manganese sequestration. Manganese ions have been shown to bind to the teichoic acid-peptidoglycan complex in Gram-positive bacteria, coordinated by carboxylates and phosphates^72^. Calcium, manganese and zinc proved to be more loosely associated with the *S. aureus* cell surface compared to magnesium, iron and copper. Cell wall-associated hydrolases, integral membrane proteins and many other enzymes require divalent cations for activity or correct folding^73–75^ and could potentially be inactivated by manganese sequestration. The inability of the chelator-resistant strains to provide resistance against the host immunomodulator CP, supports a requirement for cell wall penetration by the small-molecule chelators that is not achieved by the bulkier CP protein heterodimer. It is conceivable that direct effects on cytoplasmic membrane integrity are a consequence of the removal of stabilising metals, as previously postulated for the antibacterial properties of EDTA against Gram-negatives^16–18^.

In addition to JN206 and JN208 (Fig. 6b, c), a third chelator resistance mechanism must be manifested in the JN174/JN212 pairing (Fig. 6d). No major differences in surface charge or teichoic acid D-Ala content were apparent between these two DTPMP-selected mutants and the WT, although their thickened cell wall might credibly function as a barrier to the metal sequestering agents. An expanded peptidoglycan layer would be expected to attract metal ions through an increased number of carboxylates, exacerbated by additional negative charges from unconnected peptide cross-links. Interestingly, mutations in PBP2 in a USA300 background did appear to be slightly more negatively charged and contain slightly elevated levels of calcium. JN174 and JN212 have a slower growth rate in solid media and this could aid resistance as with other antimicrobials^76^.

A thickened cell wall and altered surface charge are a familiar means of *S. aureus* resistance against agents that target the cell envelope^77,78^. Elevated *pbp2* expression is responsible for increased cell wall thickness and is a recognised resistance mechanism in vancomycin-intermediate *S. aureus* strains^79,80^, as is reduced peptidoglycan cross-linking^81,82^. Changes in expression or mutation of *vraF* and *fmtA*, and the resulting charge modulation, are critical for resistance to a range of cationic antimicrobials, including LL-37, nisin and polymyxin B^83^. LTA D-alanylation influences the density, flexibility and permeability of the cell wall, restricting cationic antimicrobial access^84^. The prospect of modified cell envelopes harbouring a radically altered distribution of metal species is an additional feature that ought to be considered when evaluating mechanisms of antibacterial resistance.

In terms of the acquisition of resistance against EDTA and DTPMP, there were important differences between Gram-positive *S. aureus* described here and earlier experiments with Gram-negative *E. coli*^23^. Differences can largely be attributed to the presence of the impermeable outer membrane of *E. coli*, meaning that metal restriction occurs primarily in the extracellular environment. In this context, the two chelators deprived cells of different metals, meaning that resistance mechanisms depended on better utilisation of zinc, by upregulation of YeiR in the case of EDTA, or uptake of iron complexed with enterobactin via upregulation of FepA/EntD with DTPMP^23^. Notably, sequestration is predominantly affected by bioavailability and cellular accessibility rather than intrinsic affinity for metals. It is also possible that iron and zinc acquisition systems in *S. aureus* are better able to compete with metal binding chelators than their *E. coli* counterparts. Our findings present the intriguing possibility that metals targeted by chelators may vary widely between bacterial groups and offer the potential for selective elimination of problematic pathogens within microbial populations and microbiomes.

The results also highlight the potential for resistance to develop if metal sequestering agents are used at subinhibitory concentrations over a matter of weeks. These findings call into question the validity of utilising metallophores and other metal binding molecules as antimicrobials. However, encouragingly, the *S. aureus* strains isolated here showed various fitness impairments, including problems with growth, cell division, osmotic stress and increased susceptibility to the antibiotic moenomycin. Individual chelating agents or siderophores are unlikely to be employed alone as they tend to have low antibacterial efficacy^5,15^. The potential for resistance development could be overcome by employing combinations of chelators that differ in their mode of action, most appropriately in their preferences for different metal species or cellular targets. Chelators have been successfully deployed in a range of settings in concert with other antimicrobials, including antibiotics, antiseptics, and even bacteriophages^6,85,86^. The capability to penetrate biofilms is another appealing feature for the development and therapeutic utility of small molecule metallophores^87–89^. Overall, our findings reiterate and further advance the importance of cell wall architecture and surface charge in resisting antibiotics, osmotic stress, and additionally, metal sequestering agents.

## Methods

### Isolation of chelator-resistant mutants and whole genome sequence analysis

*S. aureus* strains used are listed in Table S1. EDTA (Melford) and DTPMP (Merck) were solubilised in water by addition of NaOH to pH 8. Chelator-resistant mutants of *S. aureus* FDA209P were isolated following growth at sub-MIC concentrations of each chelating agent using the same approach employed previously with *E. coli*^23^. Briefly, bacteria were initially cultivated in 1 ml of LB broth (Lennox, Sigma Aldrich) containing 0.1 mM EDTA or 1 mM DTPMP and sub-cultured daily for 15 days at 37 °C. The cultures were transferred to 96-well plates and separated by transfer of 25 μl overnight cultures into 225 μl LB broth; this allowed testing of additional (higher) chelator concentrations and were grown for 29 days. Single colonies were obtained, frozen stocks created and selected strains sequenced by MicrobesNG as described^23^. The whole genome sequence of each strain was determined, including the WT, using an Illumina MiSeq platform with 30 times coverage using a 250 bp paired end protocol. Reads were adaptor trimmed using Trimmomatic 0.30 with a sliding window quality cut-off of Q15. De novo assembly was performed on samples using SPAdes version 3.12^90^ and contigs annotated using Prodigal 2.6^91^ and Prokka 1.11^92^. A variant calling pipeline was modified from NYU genomics core using GATK^25^ as described (https://gencore.bio.nyu.edu/variant-calling-pipeline-gatk4/). Variants, including single nucleotide polymorphisms, insertions, deletions, and duplications, between the parental strains and mutant derivatives were annotated by SnpEff version 4^93^. Three additional strains isolated at 29 days were sequenced (JN207, JN209 and JN210) but contained a similar suite of mutations to JN206, JN174 and JN212, respectively, and phenotypically matched their respective partners so were not included here. All genomics sequence datasets related to this article have been deposited in the NCBI SRA database (BioProject PRJNA1186742).

### Bacterial susceptibility to chelators, antibiotics, acid and osmotic stress

For microdilution MIC assays, *S. aureus* cultures were grown in LB broth in an orbital shaker (Stuart) at 37 °C to an OD_600 nm_ of 0.07 and diluted 10-fold in LB broth for use as an inoculum^94,95^. The diluted culture (50 μl, 1 ×106 CFU/ml) was then transferred into a 96-well, round-bottomed plate (Sarstedt). Chelating agents from stock samples were diluted to yield a 2-fold series in LB broth and 50 μl mixed with the diluted inoculum to give a final volume of 100 µl. Plates were incubated at 37 °C with shaking at 130 rpm for 16 h and absorbance at OD_600 nm_ monitored on a Spectrostar Nano plate reader (BMG Labtech). Similar conditions were employed to examine sensitivity to moenomycin (Merck), although the antibiotic was also incorporated into LB agar and serial 10-fold dilutions of cultures grown to OD_600 nm_ 0.4 and 10 µl volumes applied to the surface. Sensitivity to osmotic stress was also measured in liquid culture and agar plates with LB prepared as normal at 0.1 M NaCl, or with additional salt to give 0.5 and 1 M concentrations. Polymyxin B (300 unit) discs were obtained from Thermo Scientific and applied to the surface of LB agar plates with a 0.6% soft agar overlay containing bacteria (100 µl of an overnight culture).

### Triton X-100 and lysostaphin sensitivity

In Triton X-100 autolysis assays^96^, cells were grown to late exponential phase (OD_600 nm_ ~ 0.6) in LB at 37 °C in a shaking incubator. Cells were pelleted by centrifugation and washed three times in 1 ml PBS pH 7.5 and 90 µl transferred to a 96-well plate. 10 µl of 1% Triton X-100 (Merck) was added to relevant wells and 10 µl of sterile deionised water as a control. Changes in turbidity were monitored at OD_600 nm_ at 5 min intervals at 37 °C with shaking for 3 h. Cells for the lysostaphin sensitivity assay were prepared as above and 90 µl of washed cells mixed with 10 µl of 25 µg/ml lysostaphin (Merck). Turbidity was monitored at OD_600 nm_ at 5 min intervals for 3 h using a plate reader.

### Cytochrome *c* binding assay

Association of bacteria with the positively-charged cytochrome *c* was performed essentially as described^44^. Briefly, cells were grown in LB at 37 °C in a shaking incubator to an OD_600 nm_ of 1, harvested and washed three times in 20 mM MOPS buffer. Concentrated cells were normalised to the same optical density and 5 mg/ml cytochrome *c* (Merck, C2506) added. Cells were incubated at room temperature for 15 min, pelleted and the supernatant measured at A_440 nm_ to detect unbound cytochrome *c*.

### Peptidoglycan composition

*S. aureus* cultures (20 ×40 ml; 800 ml in total) in LB broth in 50 ml screw-capped tubes, were incubated at 37 °C, with shaking at 150 rpm to an OD_600 nm_ of 0.4–0.6. Peptidoglycan was prepared as described^97^. Cells were pelleted by centrifugation and resuspended in 2 ml ice-cold 50 mM Tris-HCl pH 7.0. Suspensions were pooled and added dropwise to 120 ml of 5% boiling SDS (Melford) in a conical flask using a Pasteur pipette. Stirring was maintained at approximately 100 rpm using a stirrer bar on a hot plate. The temperature and stirring were maintained for 15 min after the final cell addition. Samples were allowed to cool to room temperature and stored at −20 °C prior to further processing. Cells were sheared using a homogeniser, and the sacculi separated for analysis by centrifugation. Wall teichoic acids and other cell wall polymers were removed using hydrofluoric acid, and purified sacculi washed in Tris-HCl to adjust the pH before a final wash was carried out in distilled water. Muropeptides were purified and analysed by high performance liquid chromatography according to protocols described previously^37,98^.

### Cell wall visualisation and measurements

An overnight culture of each strain was prepared by inoculating 5 ml of LB broth with a single colony and grown overnight at 37 °C for 16–24 h. Cells (1.4 ml) were pelleted by centrifugation and fixed with 2% glutaraldehyde in 0.1 M cacodylate (Agar Scientific). They were then exposed to 1% OsO_4_ in 0.1 M cacodylate for 30 min, washed with deionised water, followed by a series of dehydration steps at increasing concentrations of acetone from 25–100%. Cells were embedded in resin, sections cut at 100 nm on a microtome, post-stained with 2% (w/v) uranyl acetate. Samples were analysed using a Hitachin HT7800 transmission electron microscope and an EMSIS Xarosa camera. Cell wall thickness from 100 cells was measured using Fiji version 2.15.1^99^.

### Determination of cellular metal content

Fifty ml LB broth was placed in 250 ml acid-washed conical flasks prior to inoculation with *S. aureus* cells. Cultures were grown at 37 °C in an orbital shaker at 130 rpm with concentrations of EDTA or DTPMP to inhibit growth by 10–30% during the mid-log phase (~0.3–0.4 OD_600 nm_, typically 3–4 h of growth). Cell numbers were recorded using a Casy Model TT Cell Counter prior to harvesting. Cells were pelleted by centrifugation and washed three times in 10 ml of 0.5 M sorbitol, 10 mM HEPES pH 7.8. The cell pellet was then digested in 5 ml, 65% nitric acid (Suprapur®, Sigma Aldrich) for a minimum of 16 h. These digests were diluted with 2.5% nitric acid and 5.89 × 10^−4 ^μM silver standard for ICP (Sigma Aldrich) in a 1:8:1 ratio. Calibration samples were made using known quantities of metals in nitric acid (ICP multi-element standards, CertiPUR®, Sigma Aldrich & Merck) diluted in matrix-matched solution. Dilutions and a calibration curve were analysed using inductively coupled plasma mass spectrometry (ICP-MS, Thermo XSERIES 2). Instrument control, analysis and quantification were obtained using software interface PlasmaLab (Thermo Scientific) and further analysis conducted in Microsoft Excel. Mean and standard deviation values were determined from triplicate biological analyses. Results are presented in nM of metals/mg of protein. Total cellular protein measurements were made from *S. aureus* cells lysed by sonication using the Bradford assay.

### Quantification of cellular teichoic acid D-Ala content

Bacteria were inoculated in 100 ml of LB broth in a 250 ml conical flask at 37 °C with shaking at 150 rpm to mid-log phase, before cells were harvested and washed twice in 20 mM sodium acetate buffer (pH 4.8) and analysed essentially as described^46^. Resuspended cells were inactivated at 100 °C for 10 min and lyophilised to prevent loss of released D-Ala. Ten milligrams of the freeze-dried cells were resuspended in 150 µl of 0.1 M NaOH and incubated at 37 °C with shaking for 1 h. Samples were neutralised by addition of 150 µl 0.1 M HCl, pelleted by centrifugation and the supernatant lyophilised. Samples were then resuspended in 100 µl of 1 mg/ml Marfey’s reagent (1-fluoro-2,4-dinitrophenyl-5-L-alanine amide, Sigma) dissolved in 100% acetone and 25% (w/v) aqueous triethylamine. Marfey’s reagent reacts with amino acids to form diastereomeric N-aryl derivatives that can be separated by high-performance liquid chromatography (HPLC). Samples were incubated at 40 °C with shaking, neutralised with 20 µl 2 M HCl, and lyophilised. Lyophilised samples were dissolved in 1 ml of sodium acetate buffer. Separation of amino acid derivatives was accomplished using a Waters Acquity Arc UHPLC system equipped with a diode array detector at 320 nm. Samples were separated on a Waters SunFire C8 column with a flow rate of 1 ml/min by linear gradient elution from 0% to 100% acetonitrile in sodium acetate buffer. D-Ala derivatives displayed a linear relationship between the amount injected and the peak area.

### Calprotectin growth inhibition assay

Assays were performed as described^13^ with purified human CP^100^. Overnight cultures were prepared in 5 ml tryptic soy broth (TSB; Millipore) and 50 µl into 2 ml of fresh TSB in a 3 ml semi-micro cuvette (Sarstedt). Cultures were grown at 37°C with shaking to an OD_600 nm_ of 0.3 and 10 µl added to a 96-well, round-bottomed plate containing 28 µl TSB and 62 µl calprotectin buffer (20 mM Tris pH 7.5, 100 mM NaCl, 3 mM CaCl_2_). CP, in calprotectin buffer, was added at 200 and 1000 µg/ml and plates incubated at 37 °C with shaking for 16 h, with optical density readings taken every 2 h.

### Statistical analysis

For two group comparisons, such as untreated versus treated samples, a Student’s *t* test was performed. One-way ANOVA and either post hoc Dunnett or Tukey tests were employed for multiple group comparisons with an untreated control or WT. The analyses were performed in Microsoft Excel or IBM SPSS Statistics (Version 29).

## Supplementary information

Supplementary Information

## Data Availability

All genomics sequence datasets related to this article have been deposited in the NCBI SRA database (BioProject PRJNA1186742). All other data are provided within the manuscript or Supplementary Information file.
