# Supplementary material for: Enhanced resistance of metal sequestering agents by reconfiguration of the Staphylococcus aureus cell wall
Source: NPJ Antimicrob Resist. 2025 Jul 3;3:61. doi: 10.1038/s44259-025-00131-1 (PMC12229560; doi:10.1038/s44259-025-00131-1)
Supplement: Supplementary file 1 — Supplementary Information [file 44259_2025_131_MOESM1_ESM.pdf]

Supplementary Information

Enhanced resistance of metal sequestering agents by reconfiguration of the *Staphylococcus aureus* cell wall

Joy R. Paterson, Joshua M. Wadsworth, Rebecca J. Lee, Ping Hu, Jacob Biboy, Daniela Vollmer, Waldemar Vollmer, Jon Marles-Wright, Jana N. Radin, Thomas E. Kehl-Fie, Mary T. Moran and Gary J. Sharples

Table S1. *S. aureus* strains used in this study.

Strain	Source and genotype	Reference
FDA 209P / ATCC 6538P	*S. aureus* subsp. *aureus* Rosenbach; MSSA methicillin-sensitive reference strain.	(1)
JN170	Isolated from FDA 209P cultivated for 15 days in the presence of 0.1 mM EDTA	This work
JN206	Isolated from FDA 209P cultivated for 29 days in the presence of 0.1 mM EDTA	This work
JN174	Isolated from FDA 209P cultivated for 15 days in the presence of 1 mM DTPMP	This work
JN208	Isolated from FDA 209P cultivated for 29 days in the presence of 2 mM DTPMP	This work
JN212	Isolated from FDA 209P cultivated for 29 days in the presence of 1 mM DTPMP	This work
SAUSA300	JE2, USA300 LAC. Community-associated methicillin-resistant (CA-MRSA) parental strain for the NARSA collection. Obtained from the University of Nebraska *via* Kevin Waldron, Newcastle University.	(2)
SAUSA300_0959	As SAUSA300, but *fmtA*::*erm*	〃
SAUSA300_0647	As SAUSA300, but *vraF*::*erm*	〃
AH1263	LAC* (ANG1575; an erythromycin sensitive variant of USA300 LAC)	(3)
ANG4290	LAC* Δ*SAUSA300_0957*	(4)
ANG4381	LAC* Δ*SAUSA300_0957* Suppressor S1	〃
ANG4382	LAC* Δ*SAUSA300_0957* Suppressor S2	〃
ANG4384	LAC* Δ*SAUSA300_0957* Suppressor S4	〃
ANG4394	LAC* Δ*SAUSA300_0957* Suppressor S10	〃
ANG4561	LAC* *SAUSA300_1332::Tn*	〃
ANG4563	LAC* *SAUSA300_1332::Tn pbp2* SNP S2	〃
ANG4564	LAC* *SAUSA300_1332::Tn pbp2* SNP S4	〃

S. aureus genes from strain FDA209P are annotated with the prefix SAFDA_, those from USA300 with SAUSA300_. The protein sequences of FmtA and VraF from both strains are identical.

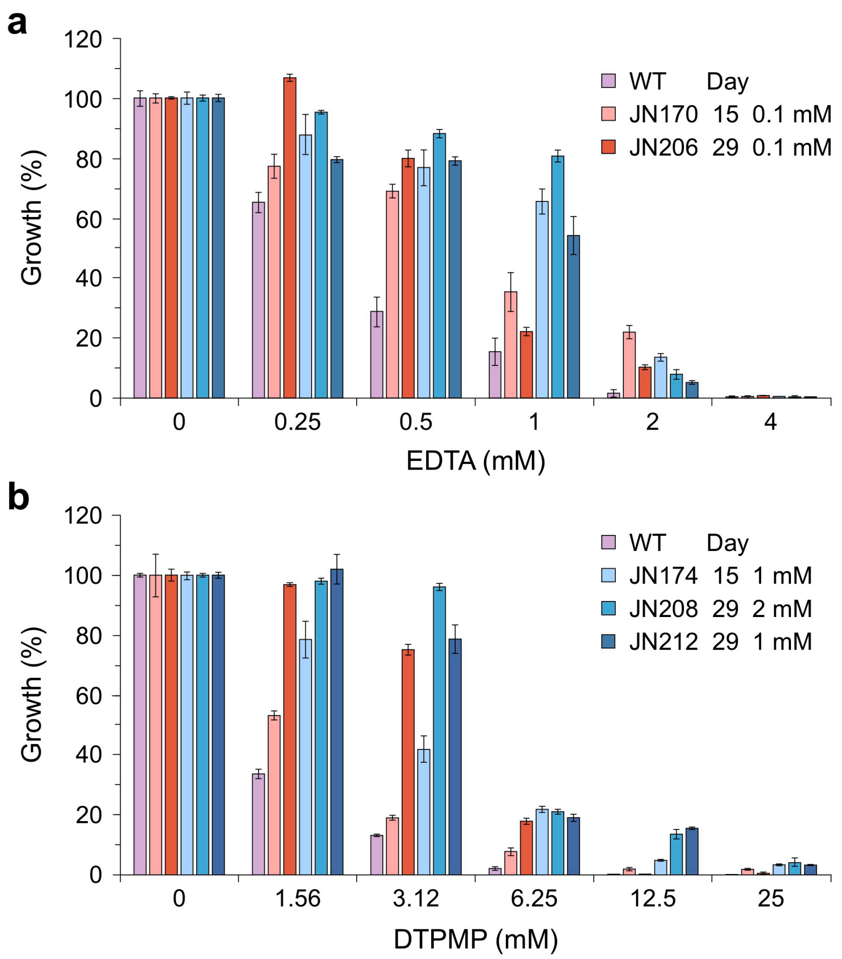

Fig. S1 | Susceptibility of *S. aureus* chelant-resistant mutants to EDTA (a) or DTPMP (b). Chelators were mixed with each strain in LB broth and incubated at 37°C for 16 h. Growth was measured at $OD_{600\ nm}$ at the endpoint and normalised against controls without chelant to give the percentage growth. Results represent the mean and standard deviation of an experiment performed in triplicate, an independent repeat of that shown in Figure 1b,c. The key in each panel shows the respective mutants isolated against EDTA (shades of red) and DTPMP (shades of blue), including the concentrations used for selection. The WT and all five chelant-selected strains were tested for resistance to EDTA (b) and DTPMP (c).

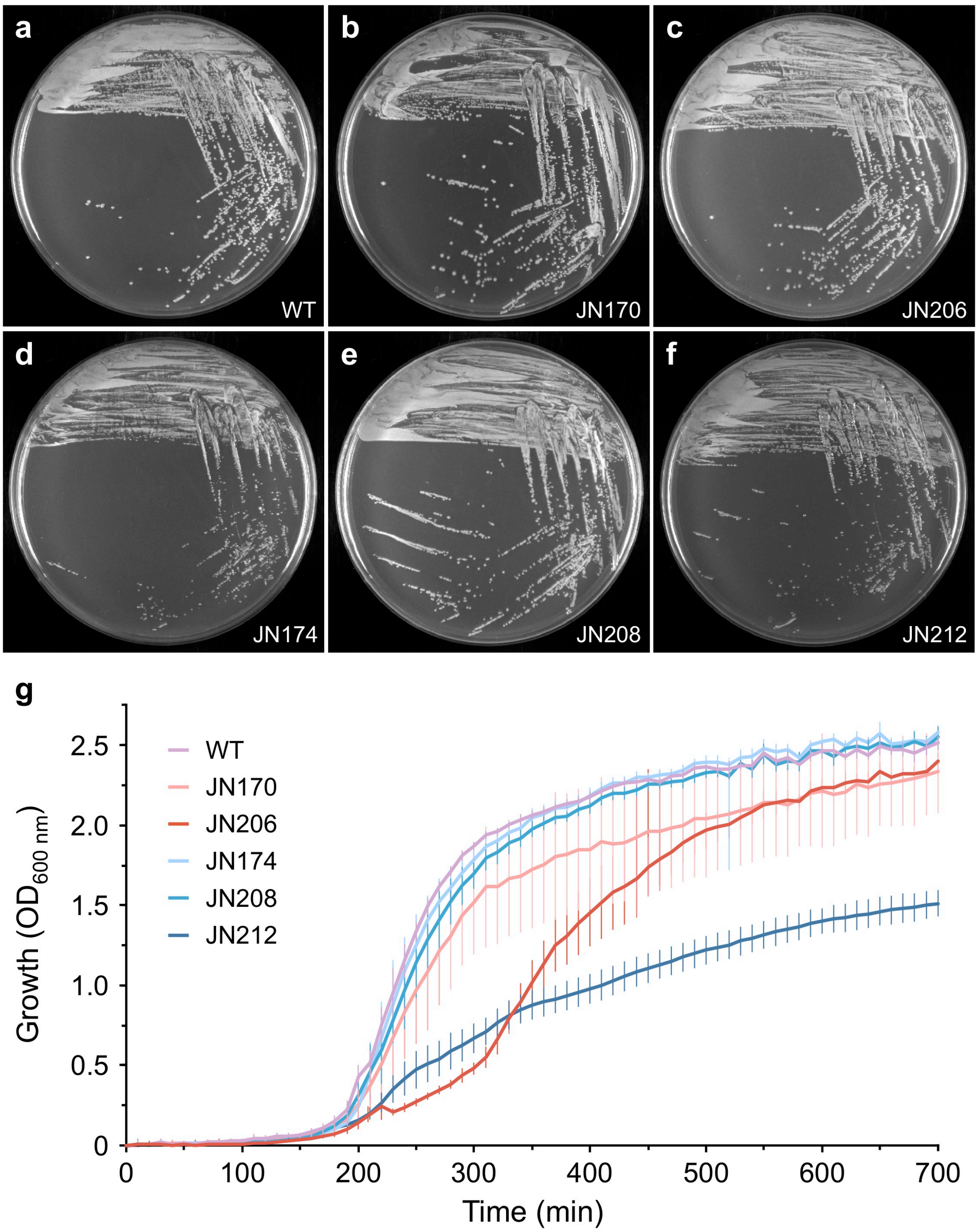

Fig. S2 | Colony morphology and growth of *S. aureus* chelant-resistant mutants. a-f Strains were streaked and grown on LB agar at 30°C for 20 h prior to imaging. **g** Growth curves of *S. aureus* chelant-resistant mutants. Strains were cultivated in LB broth at 37°C and optical density monitored at $OD_{600\ nm}$. The results are the mean and standard deviation of an experiment performed in triplicate.

		EDTA		DTPMP		
	Day isolated:	15	29	15	29	29
Gene	Function	JN170	JN206	JN174	JN208	JN212
2055	Glycine betaine transporter				A131V	
qoxA	Quinol oxidase subunit II					▲▼■
388	Lipase/esterase					T111*fs*
sasG1	Surface protein G1					E407*
sasG2	Surface protein G2	▲▼■		▲▼■		▲▼■
sbi	Immunoglobulin-binding protein				P241L	
614	AraC transcriptional regulator					H7Y
2355	Hypothetical protein					D33V
1454	Probable RNA helicase	F107Y				
butA	Oxidoreductase				A143E	
lysC	Aspartate kinase				F218Y	
citZ	Citrate synthase					▲▼■
t0048	tRNA-Ile		▲▼■	▲▼■		
r0012	23S ribosomal RNA					▲▼■
r0015	23S ribosomal RNA	▲▼■				

Membrane proteins; Surface proteins; ▲▼■ Upstream mutations

Others; tRNA and rRNA

Fig. S3 | Additional mutations identified by whole genome sequencing of *S. aureus* chelator-resistant mutants. Genes affected are grouped according to function and specific mutations listed for each strain in addition to those listed in Figure 1d. Missense mutations that change residues in the gene product are shown, replacement of an amino acid with a stop codon is indicated by an asterisk and a single mutation, affecting gene *SAFDA_0388*, is a frameshift (*fs*). Nucleotide changes upstream of genes are indicated by triangular and square symbols reflecting potential upregulation, downregulation or no effect on adjacent gene expression. Synonymous changes affecting *sdrC*, *tnpA*, *pheT*, *infB*, *gapB*, *fmtA* and *clfB* (x3) are not listed.

A relatively small number of mutations upstream of genes were identified that could potentially influence expression, although none were found in all strains indicating that they either do not contribute to chelant resistance or that they provide only a supplemental benefit. Multiple different point mutations were found in JN170, JN174 and JN212 upstream of the *sasG2* gene. SasG is a repeat protein, involved in host tissue adhesion and biofilm formation, that in some strains consists of 1637 amino acids (5). In FDA209P, *sasG* is split in two with *sasG2* lacking the N-terminal signal peptide required for export. The presence of repeat sequences may promote localised rearrangements and perhaps explain why so many mutations are located in this region. A substitution and stop codon were located at the extreme C-terminal end of *sasG1*, perhaps for similar reasons. The stop codon in SasG1 removes the last 8 residues of the protein (ELDHRSIA) which could alter the charge at the outer surface. An extra positive charge, through replacement of threonine with lysine, is added to the fibronectin binding protein FnbA (Fig. 1d) and may be important as the substitution is carried by all chelator-resistant strains. The change lies within one of

the fibronectin-binding repeats (FnBPA-1), immediately adjacent to a conserved glycine (6). Other mutations of interest, include the frameshift in gene 388, which removes the bulk of the product, a putative lipase that could be involved in cell wall metabolism. The majority of genetic alterations were missense, frameshift or nonsense mutations affecting a wide array of genes (see also Fig. 1d). Those located in a single strain, often found only in DTPMP-selected mutants, may not contribute to chelant resistance at all, but some may provide minor benefits in combination with the other changes in that strain. The mutations upstream of tRNA and rRNA genes could potentially help more generally in managing protein translation in response to growth restriction and metal starvation imposed by the chelators.

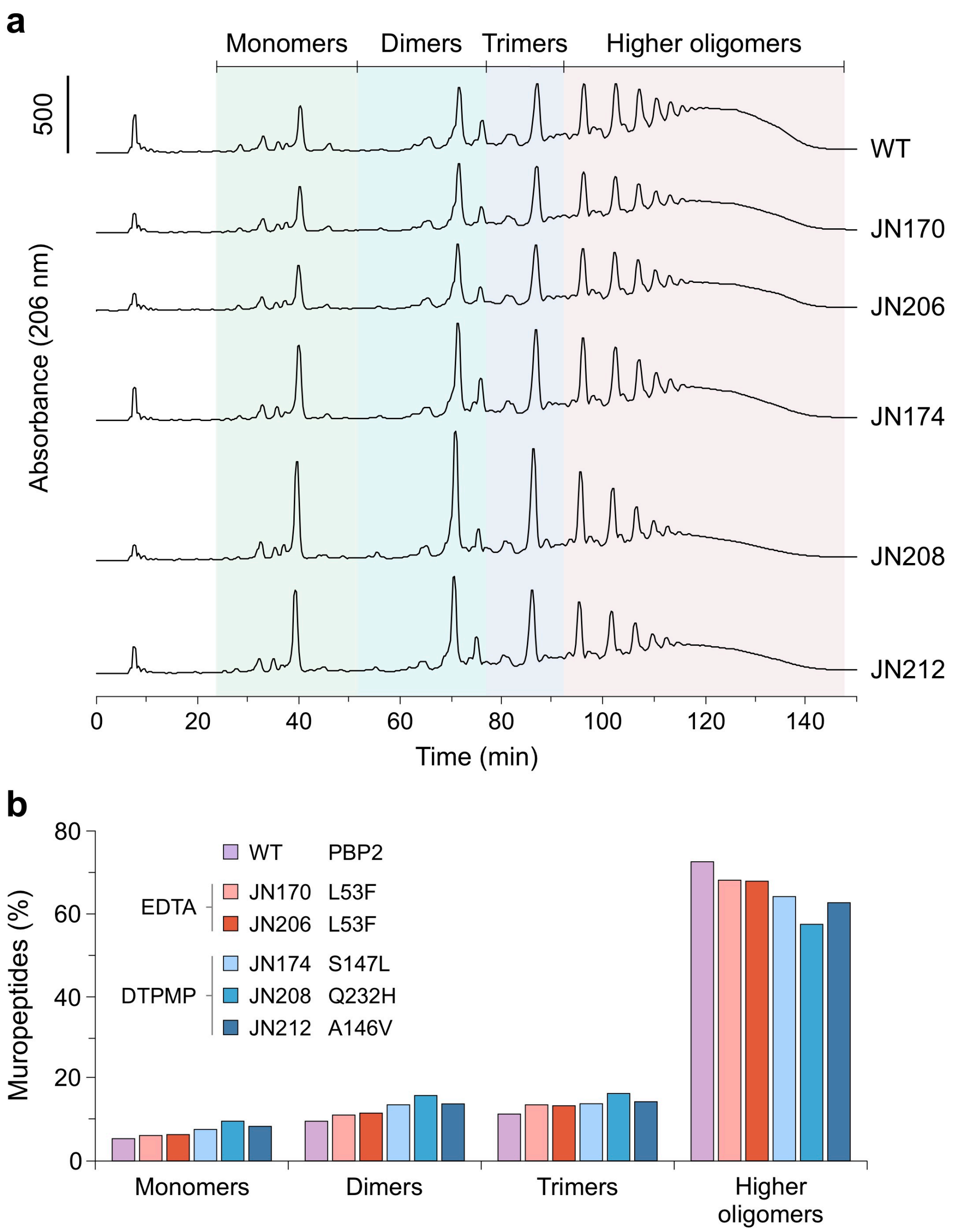

Fig. S4 | Reduced peptidoglycan cross-linking in *S. aureus* chelator-resistant mutants. a Bacteria were cultivated and peptidoglycan purified as described in the Methods. Extent of cross-linking was determined by analysis of purified muropeptides by HPLC. The data shown were obtained from an independent repeat of the experiments in Figure 2a. **b** Relative proportion of monomer, dimer, trimers and higher oligomers of cross-linked chains in WT and chelator-resistant mutants determined from the data in **a**.

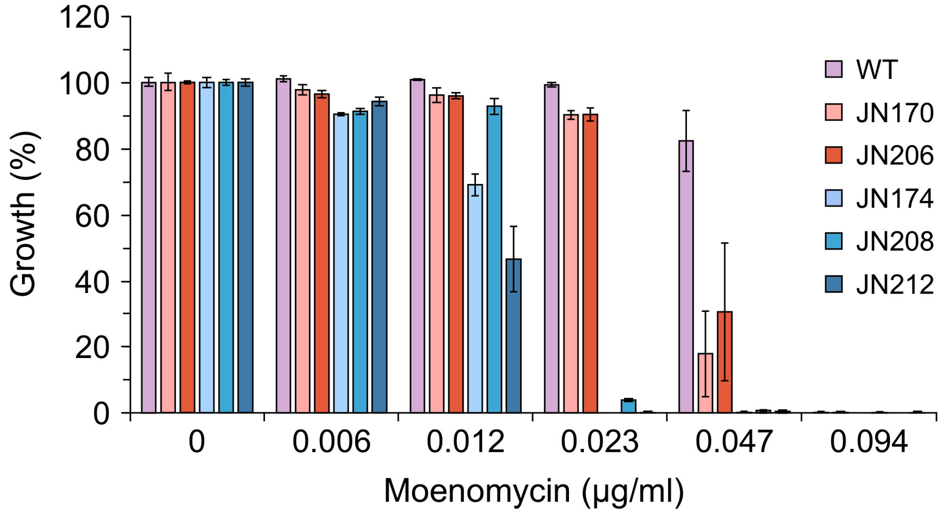

Fig. S5 | Susceptibility of *S. aureus* chelator-resistant mutants to moenomycin. Moenomycin was mixed with each strain and incubated in LB broth at 37°C for 16 h. Growth was measured at $OD_{600\ nm}$ at the endpoint and normalised against controls without antibiotic to give the percentage growth. Results represent the mean and standard deviation of an independent experiment performed in triplicate, an independent repeat of that shown in Figure 3a.

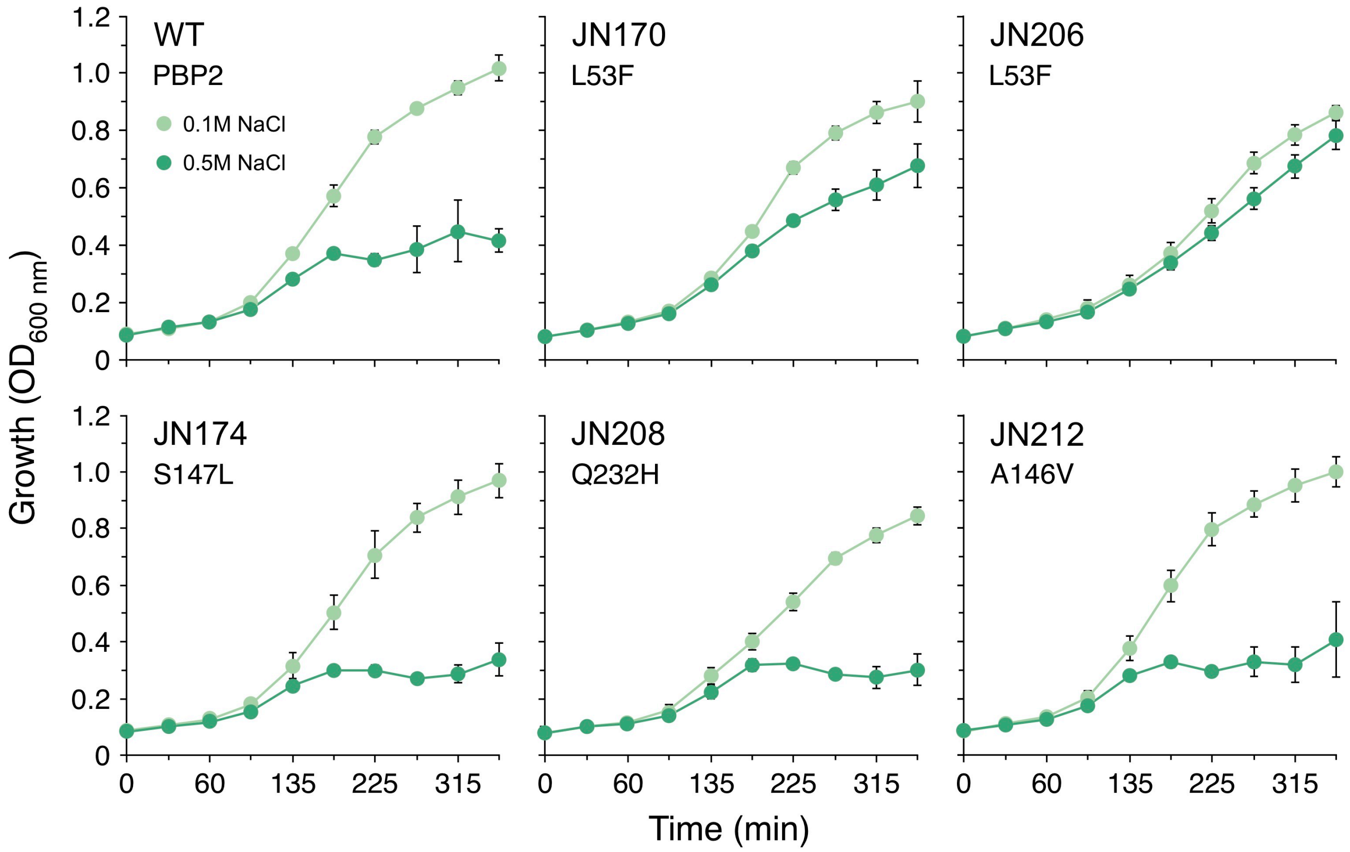

Fig. S6 | Salt resistance of *S. aureus* chelator-resistant mutants. Bacteria were cultivated in LB broth containing either 0.1 M or 0.5 M NaCl at 37°C in a shaking incubator (125 rpm) and growth monitored at $OD_{600\ nm}$. Data are the mean and standard deviation of three independent experiments. The 6 h timepoint of each experiment is reproduced from Figure 3c. The PBP2 mutations in these strains are indicated.

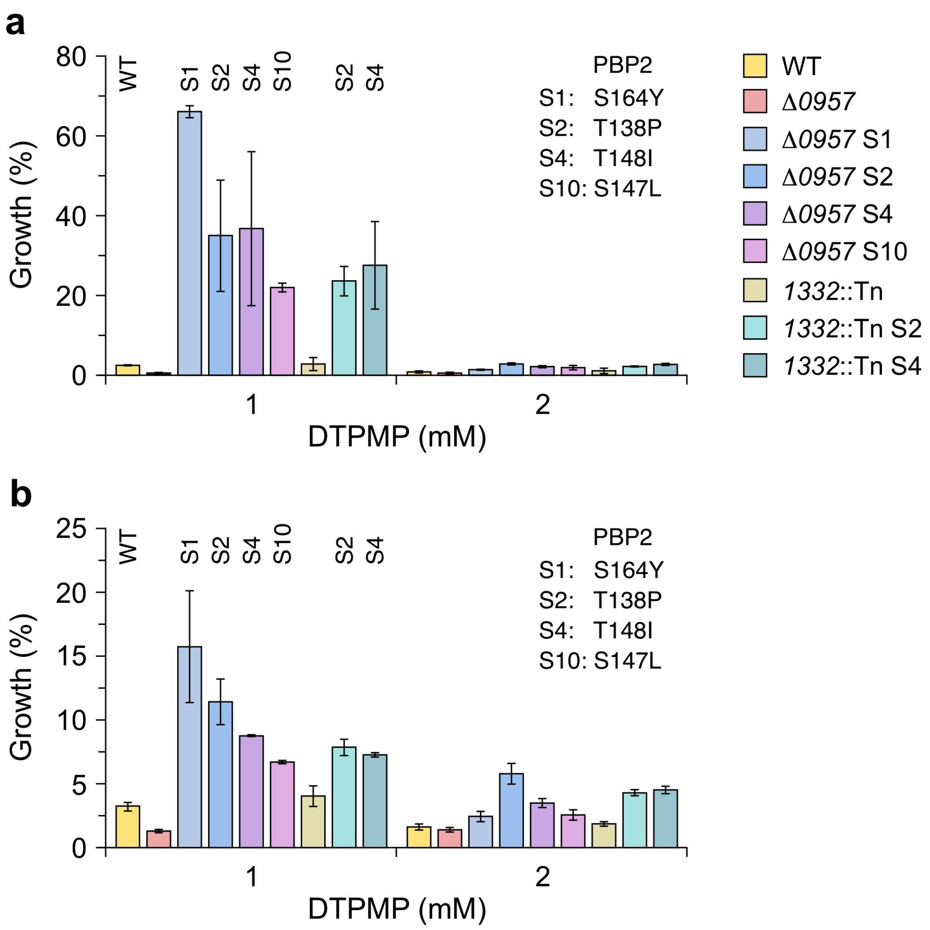

Fig. S7 | DTPMP resistance of *S. aureus* USA300 PBP2 mutants. a and **b** Two-fold serial dilutions of DTPMP were mixed with each strain and incubated at 37°C with shaking at 150 rpm for 16 h. Growth was measured at $OD_{600\ nm}$ at the endpoint and normalised against controls without DTPMP to give the percentage growth. Results represent the mean and standard deviation of two independent experiments performed in triplicate, repeats of that shown in Figure 3e. Appropriate parental controls were compared with strains carrying the different suppressor (S) mutations.

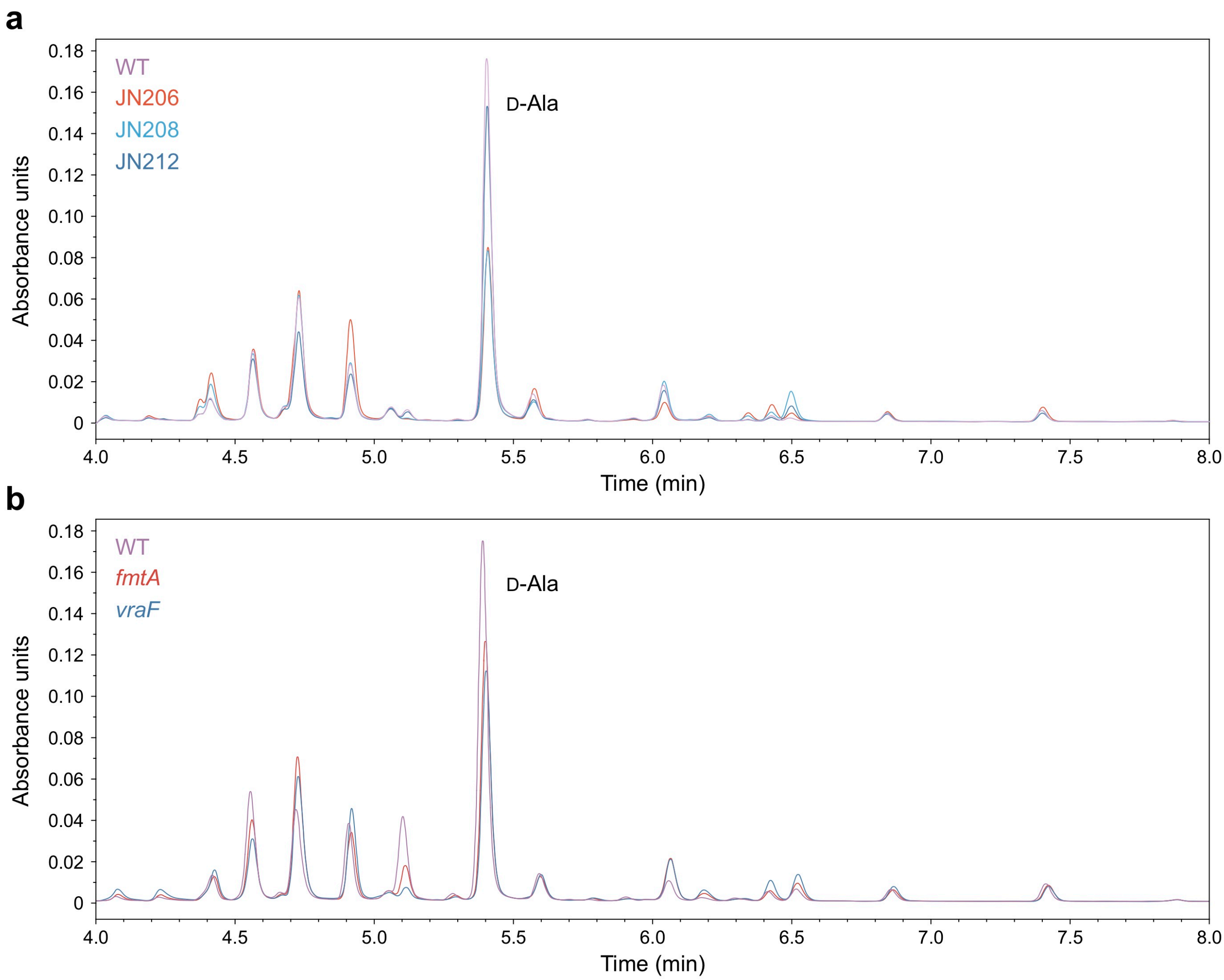

Fig. S8 | HPLC detection of D-Alanine released from whole cells. Freeze-dried *S. aureus* WT and mutant pellets were derivatized with Marfey's reagent and separated by HPLC. D-Alanine (labelled D-Ala) derivatives eluted at 5.4 min as determined by analysing a standard.

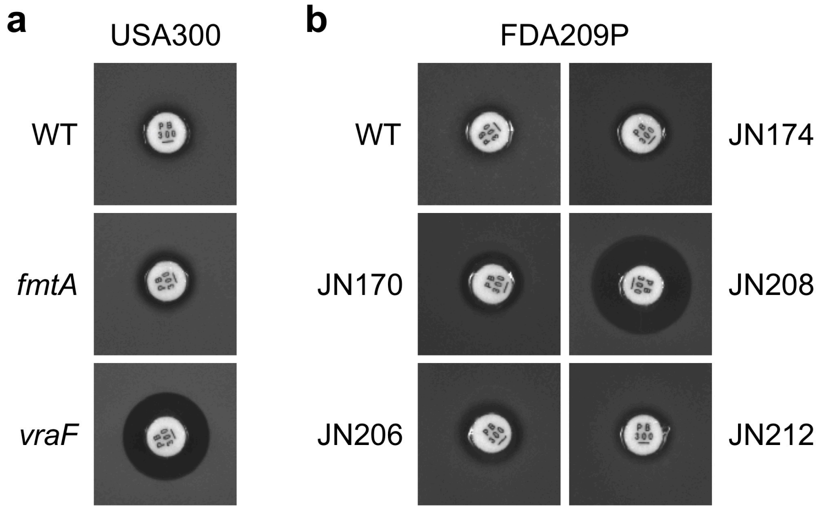

Fig. S9 | Susceptibility of *S. aureus* strains to polymyxin. Bacteria were incorporated in a 0.6% LB soft agar overlay and polymyxin B discs (300 U) applied to the surface. Plates were incubated for 16-24 h and zones of growth inhibition visualised. **a** Sensitivity of *S. aureus* USA300 WT, *fmtA* and *vraF* mutants. **b** Sensitivity of *S. aureus* FDA209P WT and chelator-resistant mutants. Representative images are shown. Three discs were placed on each plate and similar results were obtained in an independent experiment.

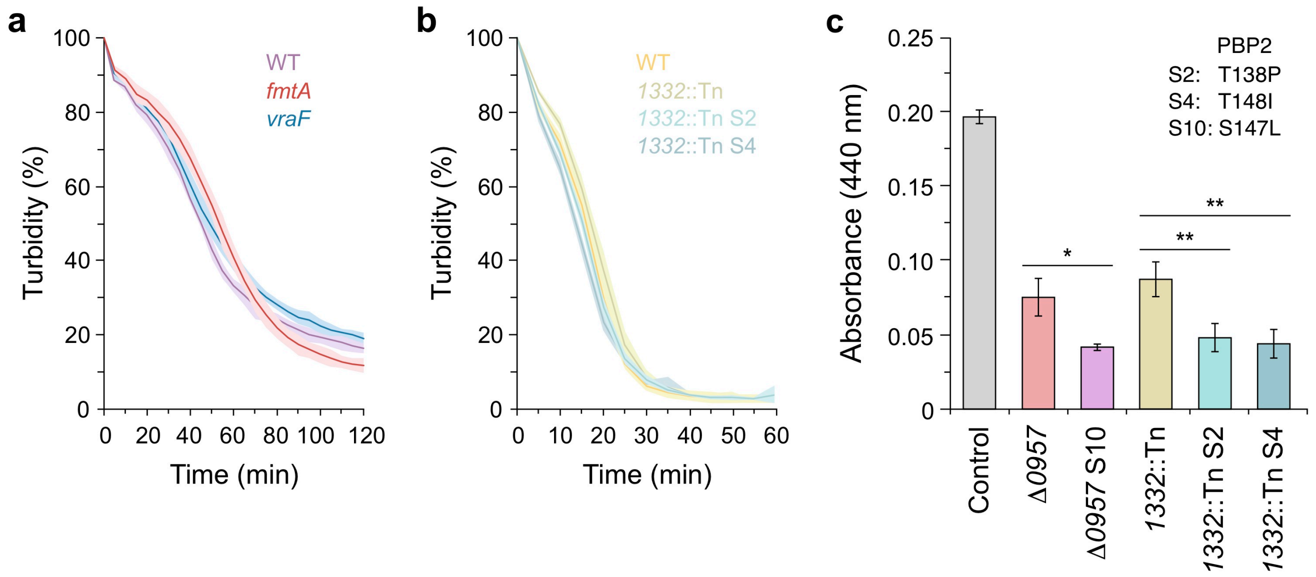

Fig. S10 | Susceptibility of *S. aureus fmtA*, *vraF* and *pbp2* mutants to lysostaphin and alterations in cell surface charge. a and **b** Sensitivity of *S. aureus* mutants to lysostaphin. Lysostaphin (2.5 µg/ml) was added to PBS-washed cell suspensions and turbidity monitored at $OD_{600\ nm}$. Results represent the mean (n = 3) with standard deviation indicated by shading. **c** Changes in bacterial surface charge of *pbp2* mutants in a cytochrome *c* binding assay. Cytochrome *c* (5 mg/ml) was added to concentrated cells and the supernatant following centrifugation to pellet cells measured at $A_{440\ nm}$ (n = 3). A student t-test was used to compare Δ*0957* and Δ*0957* S10 and one-way ANOVA analysis with post hoc Dunnett test used to compare *1332*::Tn mutant strains to the appropriate parental strain; *$P < 0.05$ and **$P < 0.01$.

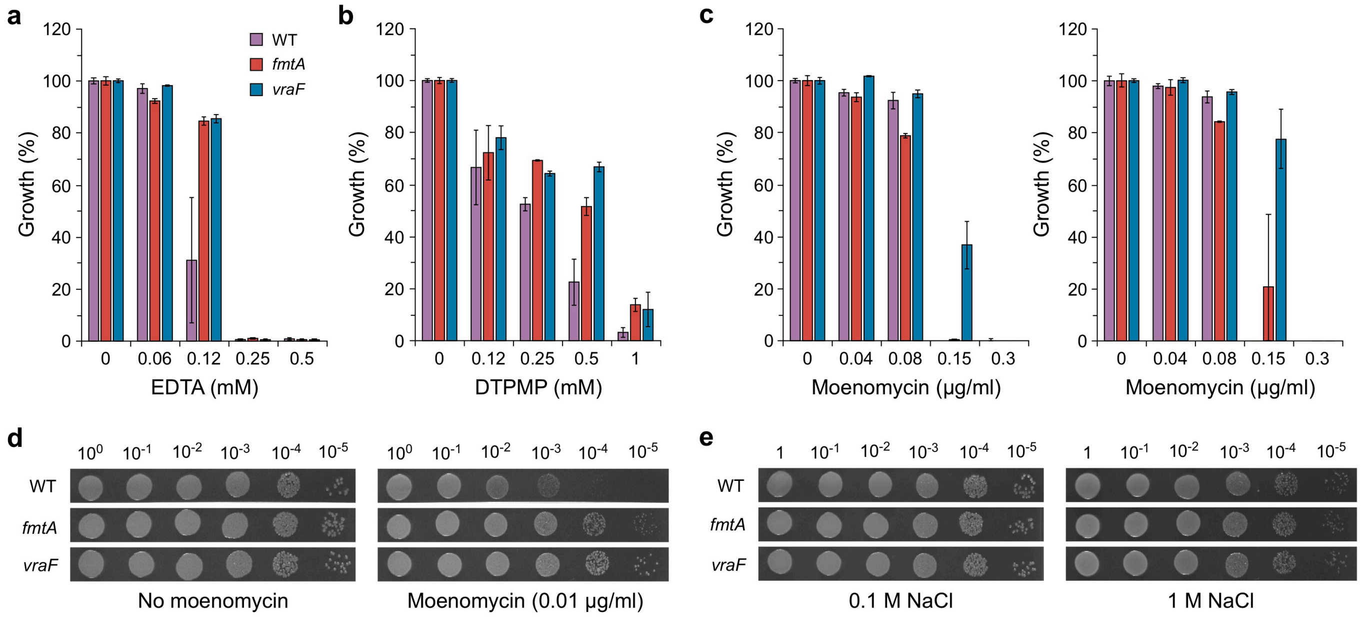

Fig. S11 | Phenotypic analysis of *S. aureus* USA300 *fmtA* and *vraF* mutants. Susceptibility of strains to **a** EDTA, **b** DTPMP, and **c** moenomycin. Two-fold serial dilutions of each chelant or antibiotic were mixed with each strain in LB broth and incubated at 37°C with shaking at 150 rpm for 16 h. Growth was measured at $OD_{600\ nm}$ at the endpoint and normalised against controls without treatment to give the percentage growth. Results in **a** and **b** represent the mean and standard deviation of experiments performed in triplicate, independent repeats of those shown in Figure 4f and g, respectively. **d** Moenomycin resistance and **e** osmotic stress resistance of *S. aureus* USA300 WT, *fmtA* and *vraF* mutants. Strains were grown to an $OD_{600\ nm}$ of 0.4, serial 10-fold dilutions performed and 10 µl volumes applied to the surface of LB agar plates in the presence or absence of moenomycin or 0.1 or 1 M NaCl. The experiments were performed three times and representative gel images are shown.

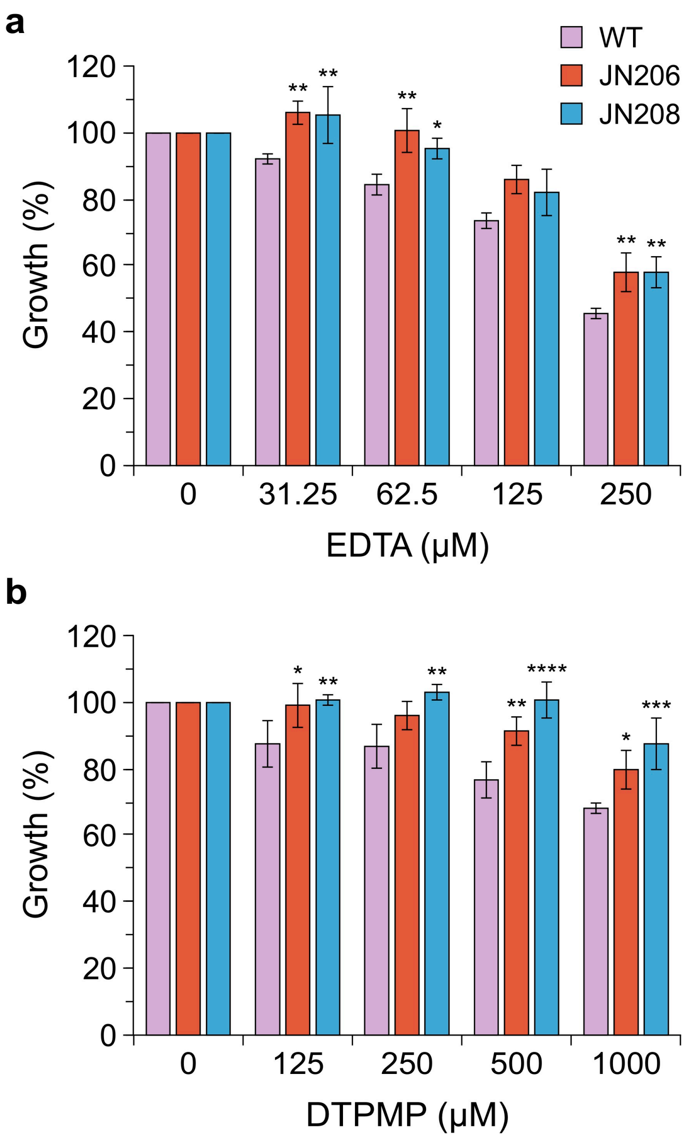

Fig. S12 | Growth inhibition of *S. aureus* by EDTA and DTPMP in larger scale cultures. Bacteria were grown in 50 ml of LB to early log-phase in a shaking incubator (125 rpm) at 37°C. **a** EDTA or **b** DTPMP was added at the outset to produce a growth inhibition of 10-30% and untreated controls set up in parallel. Data are the mean and standard deviation of 4 independent repeats (n = 4). One-way ANOVA analysis with a post hoc Dunnett test was used to compare the WT with each mutant at each concentration, *P < 0.05, **P < 0.01, ***P < 0.001 and ****P < 0.0001.

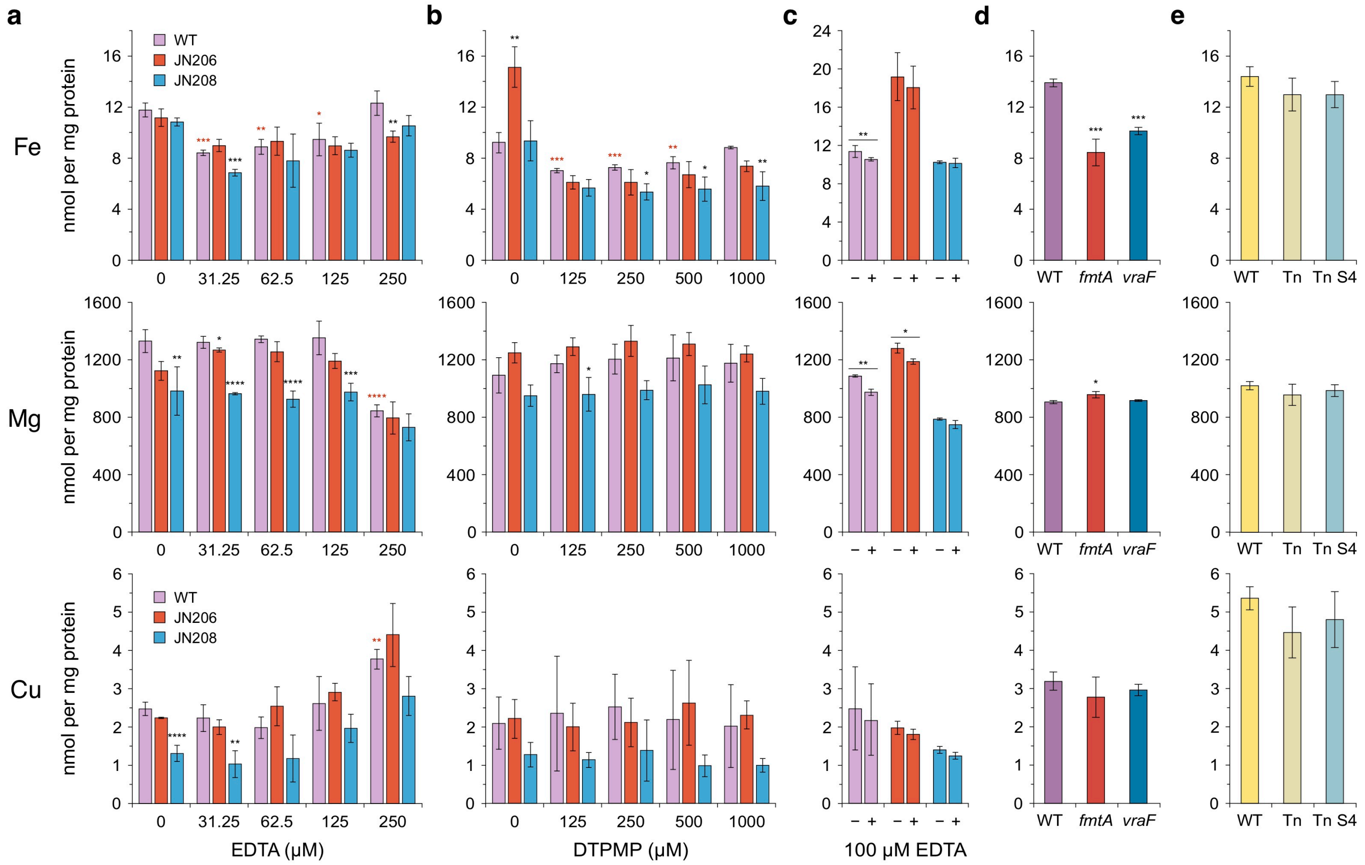

Fig. S13 | Effect of EDTA and DTPMP on the cellular Fe, Mg and Cu composition of *S. aureus* WT, JN206 and JN208 strains. Bacteria were grown in 50 ml of LB to early log-phase in a shaking incubator (125 rpm) at 37°C. EDTA or DTPMP was added at the outset to produce a growth inhibition of 10-30% and untreated controls set up in parallel. Amounts of each metal were determined by ICP-MS and presented in nmol per mg of total cellular proteins. Data are the mean and standard deviation of 3 independent experiments (n = 3). The elevated Fe seen with JN206 in panel B is due to increased growth of these samples (see Fig. S14). One-way ANOVA analysis with post hoc Dunnett test (**a**, **b** and **d**) or Tukey test (**e**) was used to compare each chelant concentration against the relevant control (n = 3). A student t-test was used to compare cells with or without EDTA in the wash buffer in **c** (n = 3). *P < 0.05, **P < 0.01, ***P < 0.001 and ****P < 0.0001. Asterisks in red (**a** and **b**) refer to comparisons between treated and untreated WT samples, those in black compare JN206 and JN208 samples against the WT under the same conditions.

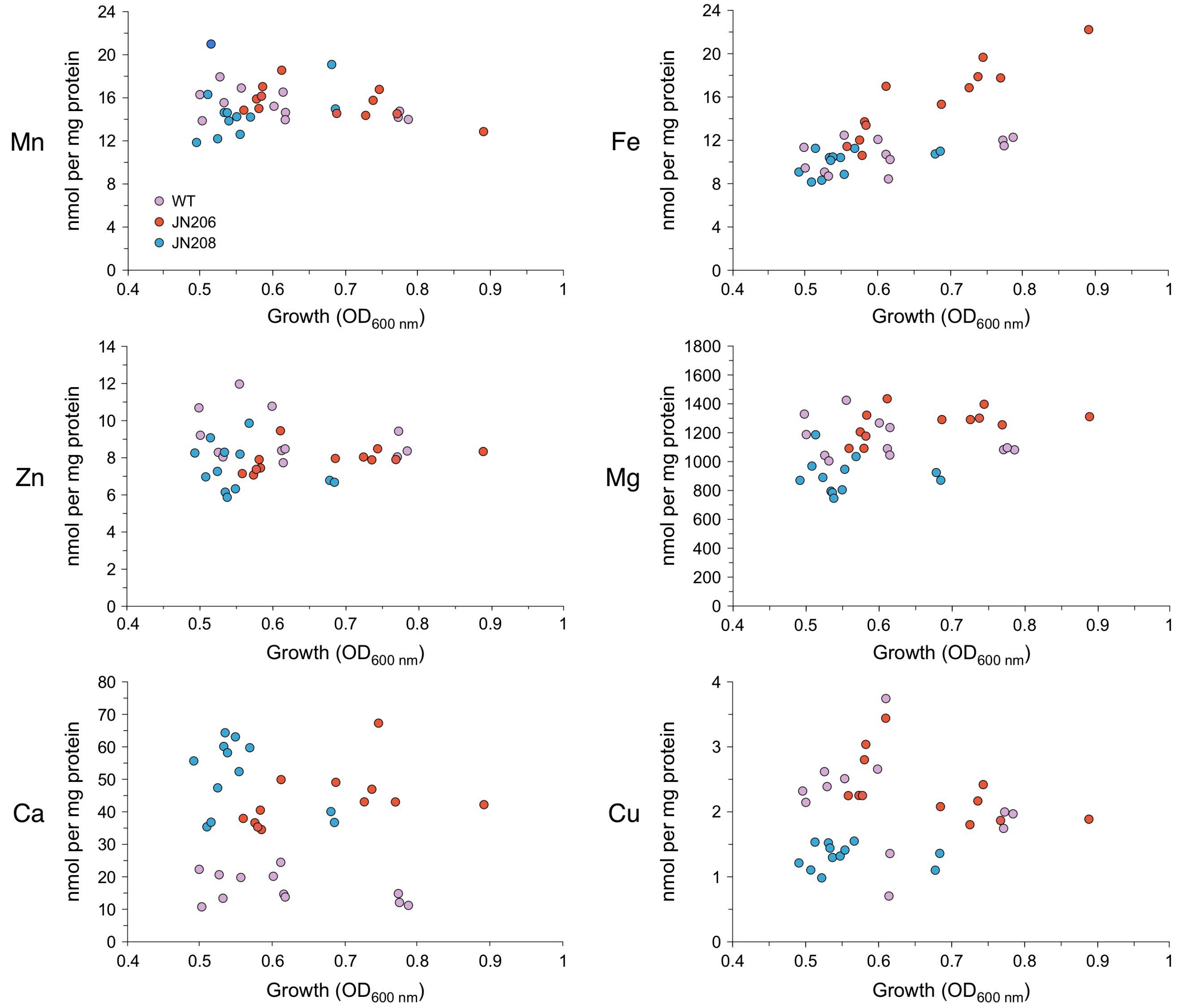

Fig. S14 | Cellular metal composition of *S. aureus* WT, JN206 and JN208 strains at different optical densities. Bacteria were grown in 50 ml of LB to early log-phase in a shaking incubator (125 rpm) at 37°C and were set up as controls in the absence of chelating agents. Amounts of each metal were determined by ICP-MS and presented in nmol per mg of total cellular proteins. Each circle represents an independent experiment (n = 12). JN206 appears able to sequester more iron than either WT or JN208 at higher optical density.

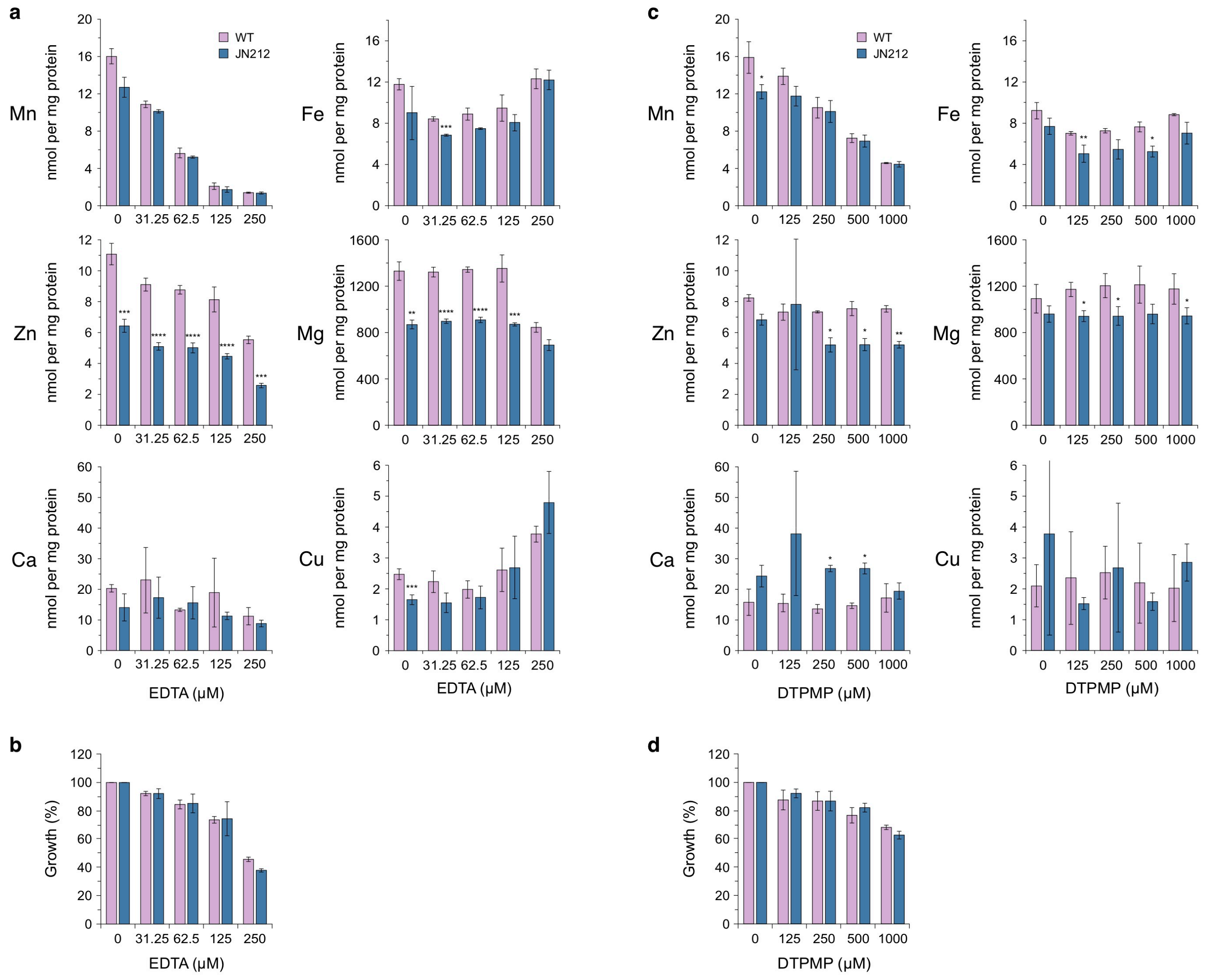

Fig. S15 | Effect of EDTA and DTPMP on the cellular metal composition of *S. aureus* WT and JN212 strains. Bacteria were grown in 50 ml of LB to early log-phase in a shaking incubator (125 rpm) at 37°C. **a** and **b** EDTA or **c** and **d** DTPMP was added at the outset to produce a growth inhibition of 10-30% and untreated controls set up in parallel. Amounts of each metal were determined by ICP-MS and presented in nmol per mg of total cellular proteins. Data are the mean and standard deviation of three independent experiments (n = 3). One-way ANOVA analysis with a post hoc Dunnett test was used to compare each chelator concentration against the relevant control, *$P < 0.05$, **$P < 0.01$, ***$P < 0.001$ and ****$P < 0.0001$. Asterisks refer to comparison between JN212 in untreated and treated samples with the WT under the same conditions. The WT values are reproduced here from Fig. 5 and Fig. S13 to facilitate comparisons.

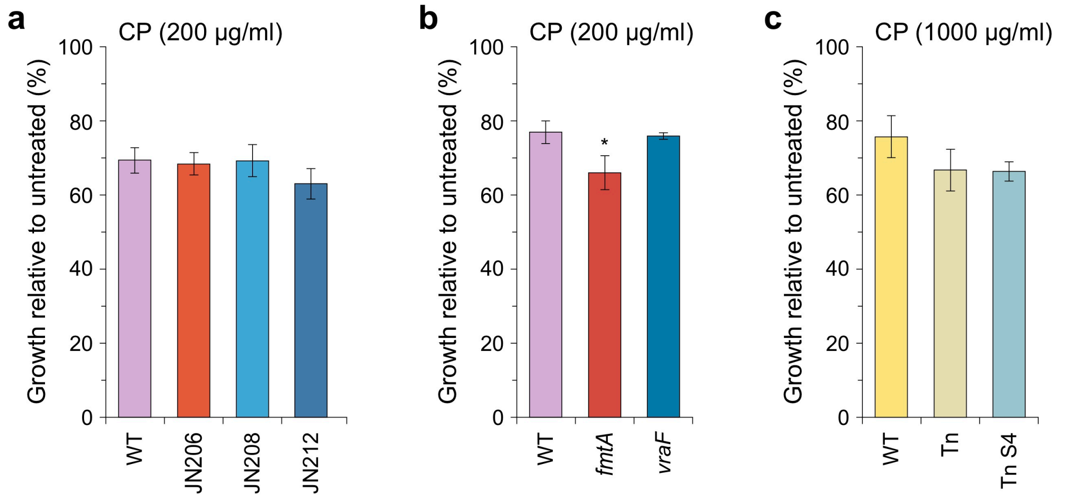

Fig. S16 | *S. aureus* mutant susceptibility to calprotectin. Early log phase cells were cultivated in TSB medium with the indicated quantities of calprotectin (CP). Growth was monitored at $OD_{600\ nm}$ over 10 h and normalised against untreated controls. Results are the mean and standard deviation of 3 independent experiments ($n = 3$) at the 6 h timepoint. One-way ANOVA analysis with a post hoc Dunnett test was used to compare each strain against the WT, *$P < 0.05$,

References

1. Singh M, Sasaki T, Matsuo M, Morimoto Y, Aiba Y, Hiramatsu K. Complete genome sequence of the drug-naive classical *Staphylococcus aureus* strain FDA209P. Genome Announc. 2015;3:e01343-15.
2. Fey PD, Endres JL, Yajjala VK, Widhelm TJ, Boissy RJ, Bose JL, et al. A genetic resource for rapid and comprehensive phenotype screening of nonessential *Staphylococcus aureus* genes. mBio. 2013;4:e00537-12.
3. Boles BR, Thoendel M, Roth AJ, Horswill AR. Identification of genes involved in polysaccharide-independent *Staphylococcus aureus* biofilm formation. PLoS One. 2010;5:e10146.
4. Schuster CF, Wiedemann DM, Kirsebom FCM, Santiago M, Walker S, Gründling A. High-throughput transposon sequencing highlights the cell wall as an important barrier for osmotic stress in methicillin resistant *Staphylococcus aureus* and underlines a tailored response to different osmotic stressors. Mol Microbiol. 2020;113:699-717.
5. Gruszka DT, Wojdyla JA, Bingham RJ, Turkenburg JP, Manfield IW, Steward A, et al. Staphylococcal biofilm-forming protein has a contiguous rod-like structure. Proc Natl Acad Sci USA. 2012;109:E1011.
6. Bingham R, Rudiño-Piñera E, Meenan N, Schwarz-Linek U, Turkenburg J, Höök M, et al. Crystal structures of fibronectin-binding sites from *Staphylococcus aureus* FnBPA in complex with fibronectin domains. Proc Natl Acad Sci USA. 2008;105:12254-8.
